# Effects of insulin and pathway inhibitors on the PI3K-Akt-mTOR phosphorylation profile in acute myeloid leukemia cells

**DOI:** 10.1038/s41392-019-0050-0

**Published:** 2019-06-19

**Authors:** Ina Nepstad, Kimberley Joanne Hatfield, Ida Sofie Grønningsæter, Elise Aasebø, Maria Hernandez-Valladares, Karen Marie Hagen, Kristin Paulsen Rye, Frode S. Berven, Frode Selheim, Håkon Reikvam, Øystein Bruserud

**Affiliations:** 1 Section for Hematology, Department of Clinical Science, University of Bergen, Bergen, Norway; 2 Department of Immunology and Transfusion Medicine, Haukeland University Hospital, Bergen, Norway; 3 Department of Biomedicine, Faculty of Medicine and Dentistry, University of Bergen, Jonas Lies vei 91, 5009 Bergen, Norway; 4 Section for Hematology, Department of Medicine, Haukeland University Hospital, Bergen, Norway

**Keywords:** Haematological cancer, Haematological cancer, Gene expression analysis, Molecular biology, Molecular medicine

## Abstract

The phosphatidylinositol 3-kinase (PI3K)-Akt-mechanistic target of rapamycin (mTOR) pathway is constitutively activated in human acute myeloid leukemia (AML) cells and is regarded as a possible therapeutic target. Insulin is an agonist of this pathway and a growth factor for AML cells. We characterized the effect of insulin on the phosphorylation of 10 mediators in the main track of the PI3K-Akt-mTOR pathway in AML cells from 76 consecutive patients. The overall results showed that insulin significantly increased the phosphorylation of all investigated mediators. However, insulin effects on the pathway activation profile varied among patients, and increased phosphorylation in all mediators was observed only in a minority of patients; in other patients, insulin had divergent effects. Global gene expression profiling and proteomic/phosphoproteomic comparisons suggested that AML cells from these two patient subsets differed with regard to AML cell differentiation, transcriptional regulation, RNA metabolism, and cellular metabolism. Strong insulin-induced phosphorylation was associated with weakened antiproliferative effects of metabolic inhibitors. PI3K, Akt, and mTOR inhibitors also caused divergent effects on the overall pathway phosphorylation profile in the presence of insulin, although PI3K and Akt inhibition caused a general reduction in Akt pT308 and 4EBP1 pT36/pT45 phosphorylation. For Akt inhibition, the phosphorylation of upstream mediators was generally increased or unaltered. In contrast, mTOR inhibition reduced mTOR pS2448 and S6 pS244 phosphorylation but increased Akt pT308 phosphorylation. In conclusion, the effects of both insulin and PI3K-Akt-mTOR inhibitors differ between AML patient subsets, and differences in insulin responsiveness are associated with differential susceptibility to metabolic targeting.

## Introduction

Acute myeloid leukemia (AML) is a heterogeneous malignancy characterized by proliferating myeloblasts in the bone marrow.^1^ Abnormal or constitutive signaling through various intracellular pathways is often observed in AML cells, including activation of the phosphatidylinositol 3-kinase (PI3K)-Akt-mechanistic target of rapamycin (mTOR) pathway.^2^ Its activation can be initiated by various mechanisms, including cell adhesion molecules, oncogenes, mutated receptor tyrosine kinases, G-protein-coupled receptors (GPCRs), and other cytokine receptors.^2^

The possible importance of PI3K-Akt-mTOR signaling in human AML has been recently reviewed.^2,3^ Although the pathway is constitutively activated in primary human AML cells, the degree of activation shows a wide range of variation among patients.^4,5^ Furthermore, the in vitro antiproliferative effect of pathway inhibitors varies among patients, and the inhibitors can even cause growth enhancement.^6^ Results of clinical studies in high-risk AML patients show that only a subset of patients show clinical responses to this treatment.^3^ Further characterization of this patient heterogeneity will be important as a scientific basis for the design of future clinical studies.

Insulin is a regulator of cellular proliferation, survival, and metabolism, and the PI3K-Akt-mTOR pathway is one of several pathways downstream of the insulin receptor^7,8^ that are involved in the regulation of these cellular functions.^9,10^ The heterogeneity of AML patients is reflected not only in in vitro and in vivo susceptibility to pathway inhibitors but also in the in vitro insulin responsiveness of primary human AML cells.^11^ Insulin is present in the bone marrow microenvironment, and experimental studies have shown that it is a regulator not only of leukemic hematopoiesis but also of normal hematopoiesis, where its effects are mediated at least partly through altered PI3K-Akt-mTOR signaling.^7,8^ In this context, we further characterized the heterogeneity of human AML patients with regard to the effects of insulin and pathway inhibitors on PI3K-Akt-mTOR activation in primary human AML cells.

## Results

### AML patients can be classified into two main subsets based on the constitutive PI3K-Akt-mTOR activation profile of their leukemic cells

We first investigated the basal or constitutive activation of the PI3K-Akt-mTOR pathway in primary human AML cells derived from all 76 patients. This examination was based on flow cytometric analysis of 10 different phosphorylation sites related to the selected pathway mediators, and the analysis confirmed our previous results, which indicated that patients fit into two main subsets characterized by generally high or generally low constitutive pathway activation (Supplementary Fig. 1).

### Insulin increases the phosphorylation of mediators in the PI3K-Akt-mTOR pathway, especially for mediators upstream of mTOR

We compared the effect of insulin at each of the 10 phosphorylation sites. For each mediator, we identified patients who showed at least 30% increased mean fluorescence intensity (MFI) in the presence of insulin compared to the corresponding medium control. Most mediators showed an increase for a large majority of the patients, but an insulin-induced MFI decrease was especially notably for three of the five phosphorylation sites downstream of mTOR, i.e., eIF4E pS209, S6 pS244, and S6 pS240 (Supplementary Table 1).

**Fig. 1:**
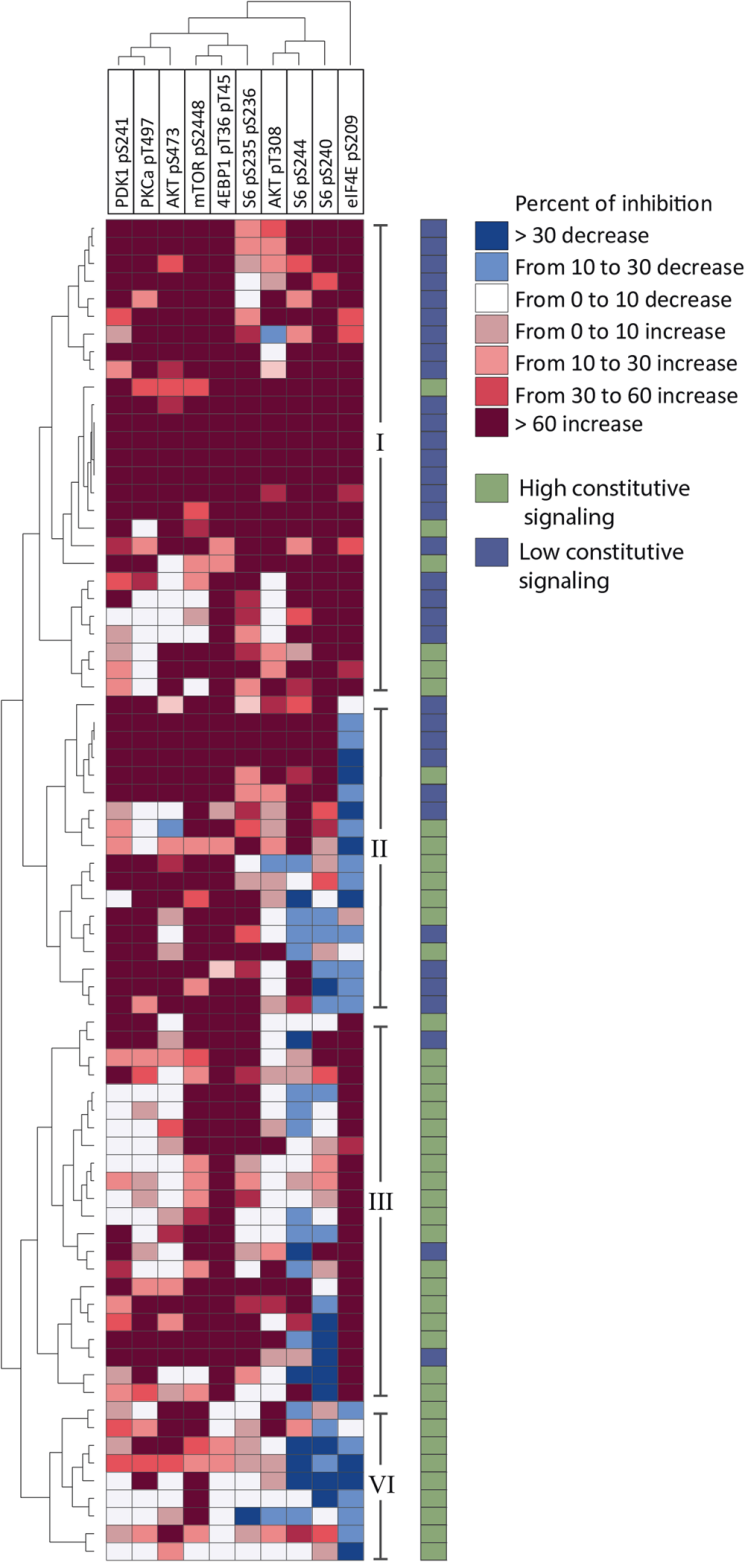
Unsupervised hierarchical clustering analysis of the effect of insulin on PI3K-Akt-mTOR activation in primary human AML cells: a study of 76 unselected patients. Patient cells from 76 consecutive patients were stimulated with human insulin (10 µg/ml) for 15 min, and their signaling profiles were analyzed by flow cytometry. The figure presents the results for analysis of relative insulin responses, i.e., the percent increase or decrease in the MFI for insulin-exposed cells compared with cells incubated in medium alone (a key to the color-coding system is given in the upper right part of the figure). Red indicates high phosphorylation/expression, and blue indicates low phosphorylation/expression. The right column shows whether the individual patients were classified as showing high (green, see the upper right part of the figure) or low (blue) constitutive PI3K-Akt-mTOR activation (see Supplementary Fig. 1). Two main clusters were identified, each including two subclusters; the subclusters are numbered I–IV

The overall phosphorylation profile for each patient was compared by calculating the relative effect of insulin on the phosphorylation for each mediator and patient, i.e., the MFI for cells incubated with insulin relative to the corresponding MFI for control cells incubated in medium alone. These relative values were analyzed using unsupervised hierarchical clustering, resulting in division of patients into two main subsets. Each of these two subsets was further divided into two minor subsets (Fig. 1, right part: minor subsets/clusters I–IV). The two main subsets (minor subsets I and II versus III and IV) differed significantly in the phosphorylation of Akt pS473 (*p* *=* 0.001), Akt pT308 (*p* *=* 0.001), mTOR pS2448 (*p* *=* 0.003), 4EBP1 pT36/pT45 (*p* *=* 0.001), and eIF4E pS209 (*p* *=* 0.010). Minor subset I showed increased phosphorylation for nearly every patient/mediator combination and differed from the other subsets (II–IV), particularly with respect to its relatively high levels of Akt pT308 (*p* *=* 0.034) and S6 p240 (*p* *=* 0.0005). Thus, minor subset I in Fig. 1 shows no evidence of a dephosphorylating effect of insulin for any mediator or patient (with only one mediator/patient combination as an exception). In contrast, the three other subsets (Fig. 1, subsets II–IV) showed decreased phosphorylation of at least one mediator, and, as expected from the data presented in Supplementary Table 1, patients in these three subclusters showed decreased phosphorylation levels for at least one of the three phosphorylation sites: eIF4E pS209, S6 pS244, and S6 pS240. These are three of the five phosphorylation sites downstream of mTOR.

### The classification of AML patients into two main subsets based on constitutive PI3K-Akt-mTOR activation is partly maintained after exposure of AML cells to insulin

The two main patient subsets identified by their phosphorylation profiles after insulin exposure differed significantly in the proportion of patients with generally low constitutive pathway activation, as identified in Supplementary Fig. 1 (27/45 in the upper main cluster versus 7/31 in the lower main cluster; *p* = 0.0013). The upper main cluster/subset shown in Fig. 1, including primarily patients who showed a strong response to insulin, includes a significant portion of the patients with low PI3K-Akt-mTOR activation prior to insulin exposure. We performed an additional principal two-component analysis based on the relative effects of insulin on pathway activation (Supplementary Fig. 2). Thus, the pre-exposure differences in constitutive pathway activation/phosphorylation still have a detectable impact on patient subclassification even after insulin exposure.

### Although AML cells with low constitutive PI3K-Akt-mTOR activation have strong insulin responses, the cells still show weak pathway activation after insulin exposure

Unsupervised hierarchical clustering analysis of the absolute MFI values of the mediators after exposure of AML patient cells to insulin divided the patients into two main clusters/subsets; the right side of the cluster showed stronger mediator phosphorylation, and the left side of the cluster showed generally weaker phosphorylation (Supplementary Fig. 3). The right subcluster, which included the patients with higher MFI values, also contained a significantly elevated proportion of patients with high constitutive pathway activation (for this classification, see Supplementary Fig. 1) compared to the left subcluster (*p* < 0.00001; see Supplementary Fig. 3, right column). Thus, the overall results show that most patients with weak constitutive pathway activation (Supplementary Fig. 1) continued to show generally weak activation after exposure to insulin, despite a relatively strong response to insulin (Fig. 1).

### Differences in the global gene expression profiles of AML cell populations with generally increased pathway phosphorylation versus populations with diverse effects

Primary AML cells derived from 30 unselected patients (corresponding to a consecutive subset) were available for a comparison of global gene expression profiles. Strong responses to insulin, with increased phosphorylation at all investigated phosphorylation sites, were found in 12 of these patients (Fig. 1, subcluster I), while 18 patients showed decreased phosphorylation of at least one mediator downstream of mTOR (eIF4E pS209, S6 pS244 or S6 pS240) after exposure to insulin (subclusters II–IV). We performed feature subset selection (FSS) analysis to identify the most discriminative genes between the two cohorts; for 175 genes, the fold change/difference between the two groups was >1.5. We performed an overrepresentation analysis to identify Gene Ontology (GO) terms that were overrepresented among these 175 genes and found 45 genes belonging to at least one GO term with an enrichment score > 20.0.

**Table 3:** The biological and clinical characteristics of the 76 AML patients included in the study

Patient characteristics			
*Age*		*Secondary AML*	
Median (years)	67	Chemotherapy	5
Range (years)	18–87	CML blast phase	1
		CMML	4
Gender		Li-Fraumeni syndrome	1
Females	34	MDS	6
Males	42	Polycythemia vera	1
		Myelofibrosis	3
De novo AML	51		
AML relapse	4	Total	21

Cell morphology			
*FAB classification*		*CD34 expression*	
M0	4	Negative (<20%)	19
M1	16	Positive (>20%)	51
M2	16	nd	6
M4	18		
M5	15		
M6	1		
M7	1		
nd	5		

Cell genetics			
*Cytogenetics* ^a^		*NPM1 status*	
Favorable	8	Mutated	24
Intermediate	6	Wild-type	39
Normal	38	nd	13
Adverse	16		
nd	8	*FLT3 status*	
		ITD	27
		Wild-type	35
		nd	14

*nd* not determined

^a^The European LeukemiaNet classification was used

After unsupervised hierarchical clustering analysis of the 45 identified genes, most strong insulin responders clustered together to the left, while most weak insulin responders clustered together to the right (*p* = 0.0011).

**Fig. 2:**
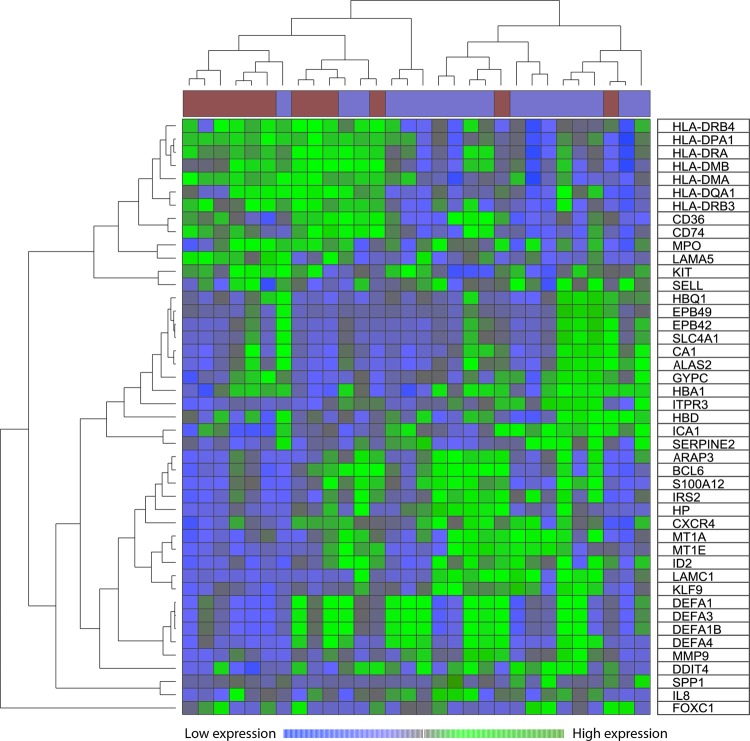
Comparison of the global gene expression profiles of patients showing a strong response to insulin and patients showing decreased phosphorylation of at least one substrate in the PI3K-Akt-mTOR pathway after exposure to insulin. The comparison included 30 unselected patients, and 45 discriminative genes were selected based on FSS analysis and overrepresentation of GO terms. Hierarchical clustering was performed by Pearson’s correlation and the weighted pair group method with arithmetic mean to measure distance. The figure shows the heat map and the corresponding dendrograms based on gene and patient clustering. The red bars at the top of the figure indicate patients with a strong effect of insulin, and the blue bars indicate decreased phosphorylation for at least one substrate. The discriminative genes are listed in the right column

The functions of the 45 identified genes were diverse (Supplementary Tables 2 and 3), and our clustering analysis identified three main gene clusters (Fig. 2). The upper cluster included mainly human leukocyte antigen (HLA) molecules, and this was the only cluster that was increased for the insulin responders. The middle cluster included several genes that are expressed in erythroid cells, and the lower cluster included genes with a wide variety of functions as well as several defensins that can be regarded as markers of neutrophil differentiation. This result indicates that insulin responsiveness was associated with differentiation.

A GO enrichment analysis of 45 differentially expressed genes was performed using online bioinformatics tools from DAVID Bioinformatics Resources 6.8, Laboratory of Human Retrovirology and Immunoinformatics (LHRI). We selected GO terms based on the criteria of (i) false discovery rate (FDR) ad modum Benjamini < 0.05 and (ii) *p-*values < 0.05. The following observations were made through analysis of cellular components, molecular function, and biological processes (see Supplementary Tables 2–4):

- We identified several highly significant terms associated with HLA class II molecules and, for some terms, CD74, including *antigen processing and presentation of peptide or polysaccharide antigen via MHC class II* and *antigen processing and presentation of exogenous peptide antigen via MHC class II* (both with *p* < 0.00001). Several significant GO terms reflected the functions of the endoplasmic reticulum/Golgi/endosomes/exosomes (Supplementary Table 4).
- We identified several highly significant terms associated with defensins, including *defense response to fungus* and *killing of cells of other organisms* (both with *p* < 0.00001).
- The three hemoglobin components were included among GO terms that differed between the two groups, but the significance was weaker (e.g., *oxygen transporter activity*, *p* = 0.012).

### Proteomic comparison of AML cells showing generally increased PI3K-Akt-mTOR phosphorylation in response to insulin versus patients with diverse effects: differential expression of metabolic regulators

We performed a proteomic comparison of AML cells that showed general increases in PI3K-Akt-mTOR activation in response to insulin (Fig. 1, subcluster I, seven patients) and those that showed diverse responses (subclusters II–IV, seven patients with decreased phosphorylation for at least one mediator in response to insulin). These patients represented patients below 70 years of age who had completed their planned intensive and potentially curative antileukemic treatment during a defined period. We used a two-sided Welch’s t-test (heteroscedastic two-sample t-test) to identify proteins with significantly different average abundance in the two groups.^12^ Proteomic analysis identified 280 proteins that differed significantly in their expression. A GO enrichment analysis of these differentially expressed proteins was performed using the online bioinformatics tools of DAVID Bioinformatics Resources 6.8, LHRI. We selected GO terms based on the criteria of FDR ad modum Benjamini < 0.005 and *p-*value < 0.05. This low FDR was chosen because the comparison was based on a relatively small number of patients in each group. We analyzed the proteins with regard to biological processes and identified 24 significantly differing GO terms (Supplementary Table 5). A large majority of the highly significant identified terms reflected metabolic differences between the two groups. These differences were also reflected in our analysis of cellular components, where the GO terms *mitochondrion*, *mitochondrial part*, *mitochondrial lumen* and *mitochondrial matrix* showed highly significant differences between the two groups (for all four terms, *p* < 0.00001 and FDR < 0.00001).

### Phosphoproteomic comparison of AML cells showing generally increased PI3K-Akt-mTOR phosphorylation in response to insulin versus those showing diverse effects: differential expression of metabolic regulators

We performed a phosphoproteomic comparison of AML cells that showed general increases in PI3K-Akt-mTOR activation in response to insulin (Fig. 1, subcluster I, seven patients) and those that showed diverse responses (clusters II–IV, seven patients with decreased phosphorylation for at least one mediator in response to insulin). The patients were the same as those described above for the proteomic studies. This comparison identified 229 proteins. Proteins that have known functions and for which phosphorylation sites showed significant differences are listed in Table 1, and a majority of them are characterized as DNA-binding proteins or involved in DNA repair and/or transcriptional regulation. Another relatively large group of proteins that are important for the regulation of translation or modulation of proteins was identified. We used GO enrichment analysis based on the differing phosphorylation sites using the online bioinformatics tools of DAVID Bioinformatics Resources 6.8, LHRI. We then analyzed the biological function of the genes (Supplementary Table 6), and the identified GO terms reflected differences in metabolism, biosynthesis, regulation of transcription, and RNA function/metabolism (all GO terms had a highly significant *p-*value and FDR < 0.005).

**Table 1:** Phosphoproteomic differences between AML cell populations/patients showing relatively strong PI3K-Akt-mTOR activation/phosphorylation in response to insulin (Fig. 1, cluster I) and populations/patients showing weaker responses (Fig. 1, clusters II–IV)

DNA, RNA, transcription and nuclear proteins	
DNA binding, DNA repair (*n* = 6)	DKC1, DDB2, SAP130, SMARCAD1, SMARCA2, MCM2
Chromatin/histones (*n* = 8)	RSF1, GATAD2B, HMGN1, SIN3A, MTA1SMARCC1, SMARCC2, H2AFY, HIST1H1B
Transcription (*n* = 26)	LYL1, ATF2, FOSL2, SIN3A, SMARCC1, SMARCC2, RSIP1, ZNF758, ZNF316, ZBTB21, POGZ, SF1, GATA2, ARID3A, PAXBP1, GTF2F1, RILP, RUNX1, DCP1A, THOC2, THRAP3, BCLAF1, MAF1, CNOT2, SNW1, HSF1
RNA metabolism and modulation; ribosomes (*n* = 20)	KHSRP, RBM17, PCFL1, SRRM1, RBMX, CLASRP, SLU7, CARHSP1, ZFP36L2, RBM15, SRSF11, CHERP, DHX16, SRF1, TRA2B, TFIP11, SRF2, SRF9, SF1, RBM12B
Other nuclear proteins (*n* = 2)	SF1, NOP58,
Nucleotides (*n* = 1)	RAPGEF6

Protein synthesis and modification	
Translation (*n* = 2)	EIF4EBP2, EIF4B,
Chaperones (*n* = 2)	NPM1, CEP170
Proteases (*n* = 11)	SMARCC1, SMARCC2, USP8, IMPDH2, TBC1D1, TBC1D10B, NRD1, CPD, SENP2, SENP3, NDRG1
Kinases/kinase inhibitors (*n* = 10)	PI4KB, NRBP1, ADRBK1, WNK1, CDK11B, CDK12, PRPF4B,
RPRD2, ZMYM4, SPAG9	
Acetylation (*n* = 2)	RSF1, SIN3A

Various proteins	
Intracellular signaling (*n* = 4)	LILRB4, PGRMC2, CHP1, APBB1IP
G proteins and associated proteins (*n* = 5)	RASAL3, SIPA1, TBC1D1, RBC1D10B, EIF5B
Cytoskeleton, microtubules,	
intracellular transport (*n* = 13)	MICAL1, FCHO1, RILP, DYNC1H1, LCP1, DSTN, DBN1, CFL1, SNX1, MAP1A, SNX1, CLASP1, CLTC
Annexins (*n* = 1)	PRKCQ
Various (*n* = 9)	TXLNA, AIFM1, DBR1 (endoribonuclease), STK11IP (enzyme modulator), TPI1 (isomerase), MECP2 (methyltransferase), VDC1 (ion channel), EFHD2 (calcium metabolism), MFAP1 (extracellular matrix)

The table is based on the PANTHER database. For clarity of presentation, the table uses gene nomenclature to identify the proteins, and the proteins/genes (indicated in the right column) are classified based on the involvement of the encoded proteins in various biological processes (left column; the number of genes/proteins is indicated in parentheses)

### A general increase in PI3K-Akt-mTOR phosphorylation in response to insulin is associated with decreased susceptibility to metabolic inhibitors

**Fig. 3:**
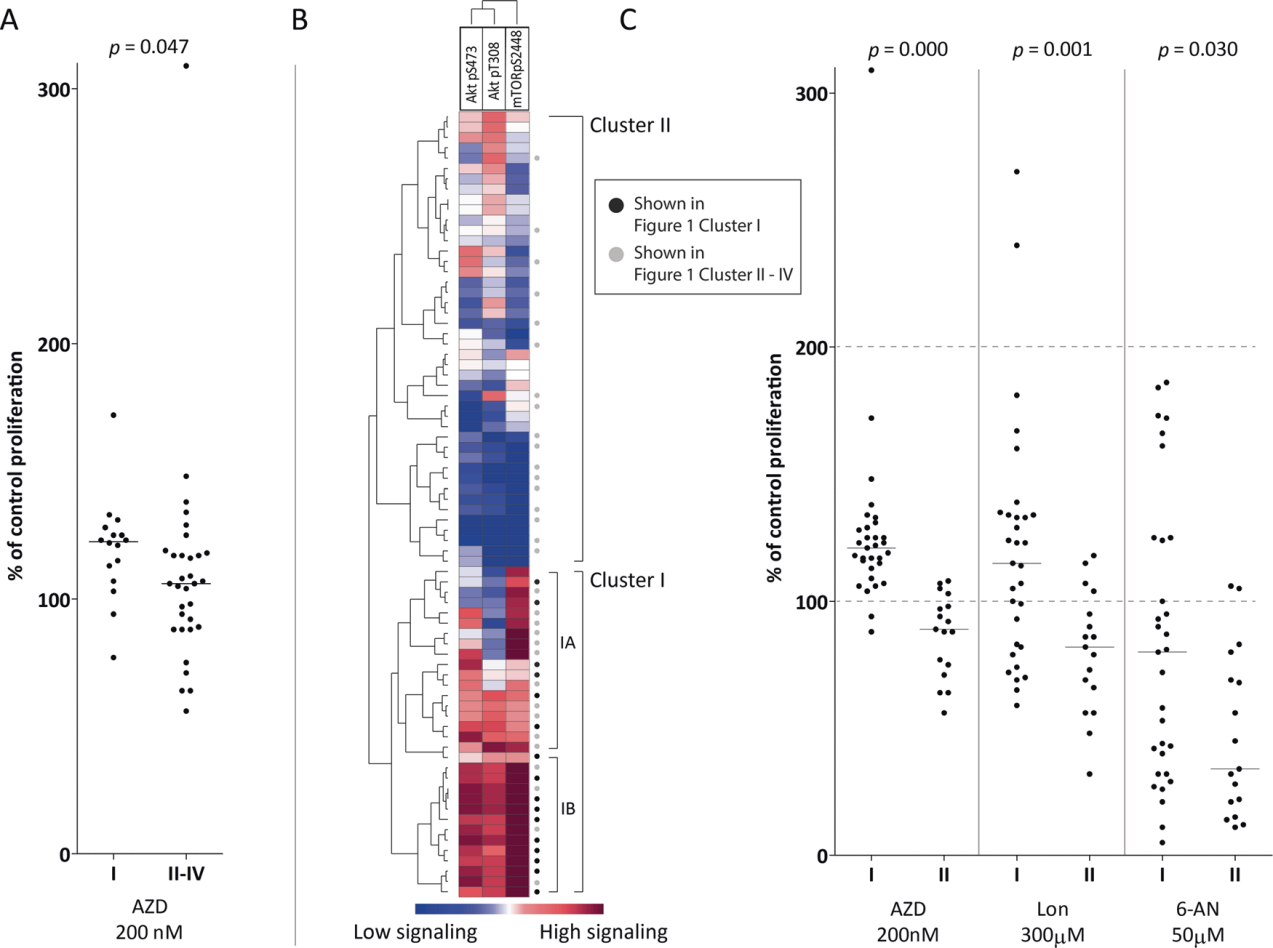
The antiproliferative effect of inhibitors of glycolysis, lactate transport and the pentose phosphate pathway in subsets of AML patients. **a** The antiproliferative effects of three inhibitors (lonidamine, AZD3965, and 6-AN) were compared for patients showing high and low insulin-induced pathway activation (Fig. 1, cluster I, versus Fig. 1, cluster II-IV, respectively). AZD3965 had a significantly weaker antiproliferative effect on AML cells, whereas the effects of the two other inhibitors did not differ significantly (data not shown). **b** A cluster analysis was performed based on the two Akt phosphorylation sites and that of its immediate downstream mediator mTOR (mTOR pS2448). Hierarchical clustering was performed by Pearson’s correlation and weighted pair group method with arithmetic mean as to measure distance. The Mann–Whitney *U*-test was used to compare different groups. Red indicates high phosphorylation/expression, and blue indicates low phosphorylation/expression. Two main clusters were identified, referred to as cluster I and cluster II. Patients (16 out of 27) from Fig. 1/cluster I (generally strong insulin effect) were included in the lower main cluster I (black dots), with subcluster IB showing the strongest insulin effect. **c** The effects of lonidamine, AZD3965, and 6-AN were measured for patients showing insulin-induced activation of Akt and mTOR (Fig. 3b, clusters I and II)

We investigated the effects of metabolic inhibitors on in vitro AML cell proliferation for a consecutive and thus unselected subset of 48 patients; 16 patients showed a general increase in pathway activation in response to insulin (Fig. 1, subcluster I), and 32 patients showed a diverse response (clusters II–IV). We tested the effects of the hexokinase inhibitor lonidamine (hexokinase being the rate-limiting enzyme in glycolysis), AZD3965 (inhibition of the lactate transporter Monocarboxylate Transporter 1, MCT-1) and 6-amino-nicotinamide (6-AN, an inhibitor of glucose-6-phosphate dehydrogenase (G6PD)/6-phosphogluconate dehydrogenase (6PGD) inhibition; G6PD is the rate-limiting enzyme in the pentose phosphate pathway). Thus, all inhibitors can mediate effects on glycolysis. We first compared the effect of these three mediators on cytokine-dependent AML cell proliferation for the two patient subsets. The antiproliferative effect of the MCT-1 inhibitor AZD3965 was significantly weaker for patients with general insulin-induced PI3K-Akt-mTOR activation than for other patients (Fig. 3a); the effects of the two other inhibitors did not differ significantly (data not shown).

Akt is an important regulator of cellular energy metabolism, including hexokinase activity.^13^ Thus, we performed a new subclassification of our patients based on a clustering analysis that included only Akt (Akt pS473 and Akt pT308) and its immediate downstream mediator mTOR (mTOR pS2448). Two main clusters were identified, and all patients from Fig. 1/cluster 1 (generally strong insulin effect) were included in the lower main cluster I and especially cluster IB (Fig. 3b). Regarding the effect of the three inhibitors for main clusters I and II (Fig. 3b), all three inhibitors had significantly weaker antiproliferative effects for patients showing strong phosphorylation of Akt-mTOR in response to insulin than for other patients (Fig. 3c). This weaker effect was observed especially for patients in subcluster IB; these patients showed significantly weaker effects than the other patients (clusters II and IA; lonidamine *p* = 0.001, AZD3965 *p* = 0.000, 6AN *p* = 0.030).

### The proteomic/phosphoproteomic differences do not reflect differences in the expression/modification of mediators downstream of the insulin receptor

Binding of insulin to its receptor can initiate downstream intracellular signaling through various pathways.^7,8^ Only 10 of the identified proteins from the proteomic studies (AYG7, APPL1, PHPT1, RAB31, PSMD9, TXNDC17, HSPD1, PARP1, SLC16A1, SIRT3) and five from the phosphoproteomic studies (EIF4EBP2, EIF4EBP1, BAD, CFL1, PRKCQ) are involved in insulin-initiated intracellular signaling, and none are involved in PDK1-Akt signaling downstream of the insulin receptor. However, spleen tyrosine kinase (SYK) protein levels were significantly increased in relatively strong insulin responders (log_2_(fold change) of 1.16, *p* = 0.026); SYK integrates several upstream signaling events, and among its downstream targets are both the PI3K-Akt-mTOR and the MEK-extracellular signal-regulated kinase (ERK) pathways, which are also downstream targets of the insulin receptor.^7,14^ Thus, differences in insulin responsiveness are associated with the intracellular context of the pathway; differential expression or phosphorylation of substrates between the receptor and Akt seem less important, although high SYK expression may contribute to strong insulin responsiveness.

### Diverse effects of the PI3K inhibitor GDC0941 in the presence of insulin: a general decrease in phosphorylation is observed only for Akt pT308 and 4EBP1 pT36/pT45

We investigated the effects of the PI3K inhibitor GDC0941 on the PI3K-Akt-mTOR phospho-flow cytometric profiles of all 76 patients, and the effects of the inhibitors were tested in the presence of insulin. We first analyzed the overall results for each of the 10 mediators separately (Table 2) and observed that GDC0941 caused a highly significant reduction in phosphorylation for most phosphorylation sites.

**Table 2:** A comparison of the phosphorylation status of PI3K-Akt-mTOR mediators in primary human AML cells after exposure to either insulin alone or insulin plus various pathway inhibitors

Phosphorylation site	Insulin alone		GDC0941			MK2206			Rapamycin		
	MFI	Range	MFI	Range	*p*-value	MFI	Range	*p*-value	MFI	Range	*p*-value
PDK1 pS241	2808	665–9286	1515	45–9137	0.000	2775	535–9243	0.882	2808	665–9287	0,482
PKCα pT497	1053	24–1399	317	48–17418	0.000	993	54–13232	0.163	1053	224–13910	0,123
Akt pS473	1171	95–11261	668	47–2790	0.001	911	78–3346	0.048	1171	95–112611	0,010
Akt pT308	643	62–3715	181	49–470	0.000	163	84–994	0.000	643	62–3715	0,000
mTOR pS2448	434	63–14860	963	43–7863	0.068	196	54–2505	0.000	434	63–14860	0,000
4EBP1 pT36/pT45	7597	98–36819	211	37–1438	0.000	228	55–1154	0.000	7597	98–368029	0,000
eIF4E pS209	1130	44–22153	361	56–1560	0.000	340	48–1249	0.000	1130	54–22153	0,000
S6 pS235 pS236	456	47–11251	239	67–1045	0.000	264	80.5–1269	0.000	457	37–11251	0,000
S6 pS244	397	70–8759	345	65–10551	0.027	322	78–8305	0.000	398	70–8759	0,010
S6 pS240	225	51–6129	100.5	68–958	0.000	108	53–1043	0.000	226	51–6129	0,000

The results are presented as the median and range for 76 patients; Wilcoxon signed-rank test, *p* = 0.005

**Fig. 4:**
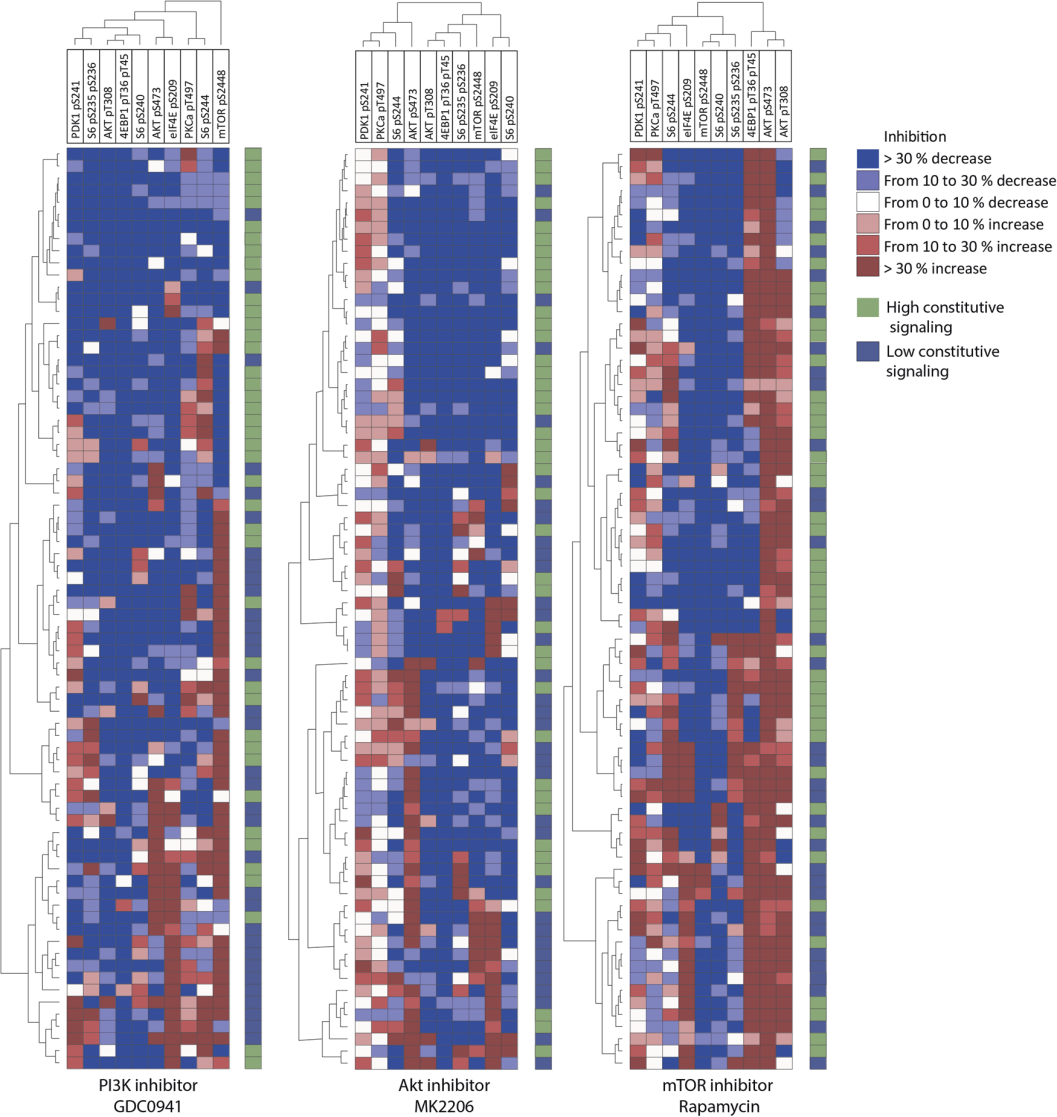
The effects of pathway inhibitors on the PI3K-Akt-mTOR activation profile of primary human AML cells. Primary AML cells derived from 76 patients were incubated with either insulin alone or insulin plus a pathway inhibitor before analysis of the activation profile by flow cytometry. The results are presented as the percent alteration of the MFI for inhibitor-containing cultures compared with the MFI for cells incubated with insulin alone. The cells were incubated with human insulin (10 µg/ml) for 15 min and thereafter with the PI3K inhibitor GDC0941 (left), the Akt inhibitor MK2206 (middle) or the mTOR inhibitor rapamycin (right). The overall results were then analyzed by unsupervised hierarchical clustering. Red indicates increased phosphorylation, while blue indicates decreased phosphorylation, in the inhibitor-containing cultures compared with the controls

**Fig. 5:**
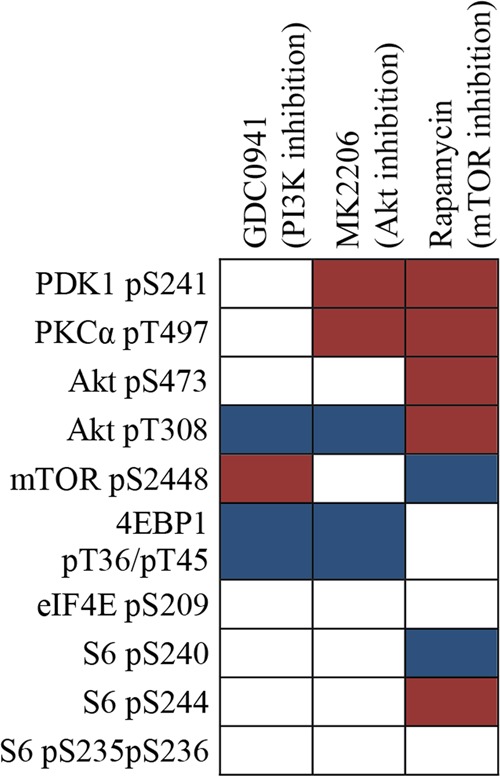
A summary of the effects of pathway inhibitors on the PI3K-Akt-mTOR activation profile of primary human AML cells derived from 76 patients. The AML cells were incubated with either insulin alone or insulin plus a pathway inhibitor before analysis of the activation profiles. The results were analyzed as the percent alteration of the MFI for inhibitor-containing cultures compared with the MFI for control cultures prepared with insulin alone. The cells were incubated with human insulin (10 µg/ml) for 15 min and thereafter with the PI3K inhibitor GDC0941 (upper), the Akt inhibitor MK2206 (middle) or the mTOR inhibitor rapamycin (lower). Blue indicates decreased phosphorylation for the corresponding mediator for at least 66 of the 76 patients, white indicates divergent effects and red indicates increased or unaltered phosphorylation for at least half of the patients. All inhibitors were added at a final concentration of 100 nM

We further analyzed patient heterogeneity and the effects of GDC0941 on the PI3K-Akt-mTOR profile for individual patients in the presence of insulin by comparing primary human AML cells incubated with insulin alone and those incubated with insulin plus GDC0941 (Fig. 4 left, Fig. 5). We compared the relative MFI for all patients and substrates, i.e., the MFI for cultures with GDC0941 plus insulin relative to the MFI for cultures with insulin alone. Unsupervised hierarchical clustering analysis showed a strong decrease in Akt pT308 and 4EBP1 pT36/pT45 phosphorylation among most patients in all subclusters. The response of mTOR pS2448 varied significantly across patients; a subset of 29 patients formed a separate upper cluster in which most patients showed decreased mTOR p2448 phosphorylation. Most of the patients in this cluster also showed generally high constitutive pathway activation, as defined in Supplementary Fig. 1 (24/29 versus 18/47 patients in the other clusters, *p* = 0.00015). Divergent effects were observed for the other mediators. In the presence of insulin, GDC0941 caused a predictable inhibition of two pathway mediators, whereas its effects on the other mediators differed and were partly dependent on original constitutive pathway activation.

### Diverse effects of the Akt inhibitor MK2206 in the presence of insulin: increased phosphorylation of upstream PDK1 and PKCα pT497 and generally decreased phosphorylation only of Akt pT308 and 4EBP1 pT36/p45

We investigated the effects of the Akt inhibitor MK2206 on the PI3K-Akt-mTOR phosphorylation profile in the presence of insulin for all 76 patients. When analyzing the overall results for each individual mediator, we detected significant decreases in phosphorylation for most of them (Table 2).

We analyzed the effects of MK2206 through a procedure similar to the one we used for GDC0941, performing a comparison of phosphoproteomic profiles after incubation of primary human AML cells in insulin with and without MK2206 (Fig. 4 middle, Fig. 5). The effects are presented as the relative responses (i.e., MFI for insulin plus MK2206 versus MFI for insulin alone). For almost all patients, we observed unaltered or increased phosphorylation of the upstream mediators PDK1 pS241 and PKCα pT497 together with decreased phosphorylation of Akt pT308 and 4EBP1 pT36/pT45. Patients were separated into two main subsets; the upper 26 patients clustered together and showed a strong decrease in the phosphorylation of most mediators not already discussed and an increased frequency of patients with high constitutive phosphorylation, as defined in Supplementary Fig. 1 (21/26 versus 29/50, *p* = 0.00015). Thus, similar to the PI3K inhibitor, the Akt inhibitor showed the general effects common to most patients for a limited number of the pathway mediators but showed increased phosphorylation of the upstream PDK1 pS241 and PKCα pT497 (Fig. 5). For the other mediators, we observed a divergence that seems to partly reflect constitutive phosphorylation.

### Diverse effects of the mTOR inhibitor rapamycin in the presence of insulin: generally decreased phosphorylation is observed only for mTOR pS2448 and S6 pS240, whereas upstream mediators and especially Akt p308 are associated with increased phosphorylation

We investigated the effects of the mTOR inhibitor rapamycin on the PI3K-Akt-mTOR phosphorylation profile in the presence of insulin for all 76 patients. When analyzing the overall results for each individual mediator, we detected a significant decrease in phosphorylation for most of them (Table 2).

We investigated the phospho-flow cytometric profiles of primary human AML cells after incubation in insulin with and without rapamycin (Fig. 4 right, Fig. 5). As with the Akt inhibitor MK2206, the upstream mediators Akt pS473 and especially Akt pT308 were associated with unaltered or increased phosphorylation for most patients, and we observed a significant decrease in phosphorylation of mTOR pS2448 and S6 pS240. In contrast to the patterns of the two other pathway inhibitors, there was no decrease in phosphorylation for S6 pS244 (Fig. 5). Other downstream substrates showed diverse effects, although decreased phosphorylation was most common. The divergent effects of this inhibitor showed no association with the constitutive pathway activation classification based on the clustering presented in Supplementary Fig. 1 (Fig. 4, right part, see the column to the right).

### Insulin generally increases PI3K-Akt-mTOR activation, and none of the pathway inhibitors causes a complete reversal of these insulin effects

We investigated whether pathway inhibitors caused a partial or complete counteraction of insulin-induced increases in mediator phosphorylation/activation back to the constitutive level. We used hierarchical clustering analysis to compare the MFI for cells incubated with insulin plus an inhibitor (see Fig. 4) and the MFI for corresponding medium controls (i.e., the constitutive phosphorylation, see Supplementary Fig. 1). Although the effects of the pathway inhibitors varied for inhibitors and mediators, all of the investigated pathway inhibitors caused only a partial reversal of the effect of insulin (Supplementary Fig. 4).

## Discussion

Insulin stimulates proliferation of primary human AML cells for at least a subset of patients,^11^ and the PI3K-Akt-mTOR pathway is one of several pathways that may be activated by the insulin receptor.^7,8^ We observed that insulin increased the phosphorylation of several members of this signaling network, but insulin responsiveness and the effect of pathway inhibitors varied across patients.

We investigated primary human AML cells derived from patients with high relative and/or absolute levels of AML cells in peripheral blood, allowing highly enriched AML cell populations to be prepared by a simple and highly standardized method of gradient separation alone.^15,16^ As discussed in detail previously,^15^ our samples include at least 90% AML blasts and often more than 95%, and by using gradient separation alone, we can achieve highly standardized cell preparation with a minimal risk of inducing the functional alterations described for more extensive separation procedures. The contaminating cells are mainly lymphocytes; these cells can release several hematopoietic growth factors, but this release requires specific activation^17^ that does not occur during the short incubation periods that we used. Furthermore, our previous studies showed that patients selected in this way are representative of AML in general with regard to cytogenetic analysis and *FLT3* abnormalities, the most important genetic markers for prognostication and in vivo chemosensitivity.^18^ Leukemic blood cells are sufficiently representative of corresponding bone marrow AML cells; although there are quantitative differences, the major characteristics are comparable.^4^ Finally, our methods for cryopreservation and thawing are highly standardized. However, the cells have decreased viability after thawing^19^ and undergo spontaneous apoptosis during culture. Nevertheless, most patient samples have a viability of approximately 70%, with exceptional patients having higher or lower viability. Our incubation periods in this study are so short that spontaneous in vitro apoptosis will not reduce the viability further. Cryopreservation may reduce the expression of cell surface molecules;^20^ however, there are several advantages of using cryopreserved cells, including the ability to analyze several patient samples in the same experiments with the same batches of reagents and to reanalyze the same patient samples in follow-up experiments (e.g., to document reproducibility).

PI3K-Akt-mTOR can be regarded as a signaling network rather than a single pathway.^3^ In our present study, we investigated signaling through the main track of the pathway, from the upstream PDK1 to several substrates downstream of mTOR. This pathway was the basis for our selection of mediators.

Insulin, insulin-like growth factor 1 (IGF-1) and IGF-2 are closely related mediators. First, the insulin receptors are homodimers of receptor A or B chains (IR-A, IR-B), and the IGF-1 receptor (IGF-1R) is a homodimer.^21,22^ However, the homodimeric IGF-1R receptor can also bind to IGF-2 (although with lower affinity) and insulin (with very low affinity); the IR-A homodimer binds to all three ligands, and the IR-B homodimer binds to insulin and IGF-2.^21,22^ Second, the receptor chains can form heterodimers (IGF-1R with IR-A or IR-B), and the IGF-1R/IR-A heterodimer binds to all three ligands, although only insulin and IGF-1 bind with high affinity.^21,22^ Third, both systems initiate downstream signaling through PI3K-Akt and RAS-MEK-ERK.^21,22^ Human AML cells express both IR-A and IGF-1R;^23^ crosstalk between the two systems will thus take place at both the receptor level and the downstream signaling level.^21^ Finally, autocrine IGF-1/IGF-1R stimulation seems to contribute to the constitutive activation of PI3K-Akt-mTOR in primary human AML cells,^23^ and the mTOR-mediated feedback on Akt phosphorylation seems to involve insulin/IGF-mediated signaling upstream of Akt.^3,23^ Taken together, these observations suggest that previous constitutive IGF-1 release contributes to constitutive PI3K-Akt-mTOR activation in primary AML cells, but the additional patient heterogeneity after incubation with insulin is probably caused by insulin alone because the incubation period is most likely too short to induce increased autocrine stimulation by increased IGF-1 synthesis and release.

Primary human AML cells express insulin and IGF receptors,^23^ and a recent article described associations between pretreatment serum levels of various IGF binding proteins and between the serum fraction of bound IGF and a favorable prognosis with increased survival.^24^ Systemic insulin/IGF levels thus seem important for the effect of insulin/IGF1 in the AML cell microenvironment, and experimental studies further suggest that both insulin^25,26^ and IGF^27^ have growth-enhancing and/or antiapoptotic effects in primary human AML cells. However, the heterogeneity of constitutive and insulin-induced PI3K-Akt-mTOR network phosphorylation in primary AML cells has not been addressed previously, and our present study shows that constitutive phosphorylation (at least partly IGF dependent) differs between patients, but exposure to insulin adds further heterogeneity. Our present study is also the first to compare the proteomic and phosphoproteomic characteristics of patient subsets with different pathway activation/responsiveness to insulin, suggesting that metabolic regulation differs between these subsets. Metabolic markers have prognostic value in AML,^28^ and metabolic targeting is considered a possible therapeutic strategy.^29^ Our present observations suggest that the antileukemic effects of various strategies for metabolic targeting differ between patient subsets identified by analysis of PI3K-Akt-mTOR activation. The combination of PI3K-Akt-mTOR inhibition and metabolic targeting is now under investigation for other cancers,^30,31^ and our results suggest that this combined strategy should be further considered in AML, but possibly only for selected subsets of patients. Pretherapy transcriptomic or proteomic analyses of AML cells may be used to identify the subsets.

Immature leukemic stem cells are regarded as responsible for chemoresistant AML relapse. However, several reports suggest that characteristics of the total AML cell population reflect key characteristics of the immature minority of AML cells in the hierarchically organized AML cell populations. Genetic abnormalities are common for a majority of cells within the AML cell population.^1^ The prognostic impact of mRNA profiles (e.g., leukemic stem cell profiles),^32^ epigenetic regulation,^33^ single molecular markers detected at the mRNA^34^ or protein level,^35^ and constitutive cytokine release profiles^36^ has been demonstrated through analysis of the overall AML cell population. Finally, in vivo evaluation of chemosensitivity by examination of bone marrow blast counts 17 days after the start of induction therapy and the use of minimal residual disease estimation are based on evaluation of common AML cell characteristics.^35^ Thus, the biological characteristics of the total AML cell population reflect the in vivo chemosensitivity of AML.

We used 10 µg/mL insulin corresponding to a concentration of 1700 nM. In vivo insulin levels can be as high as 2.8 nM after glucose intake.^37^ The concentration used in previous studies of insulin enhancement of in vitro AML cell proliferation was 1–10 μg/ml.^11,38^ Our selected insulin concentration and time of insulin exposure were based on pilot studies of the effects of insulin on PI3K-Akt-mTOR activation in primary human AML. The insulin/IGF system is a regulator of both normal^39^ and leukemic hematopoiesis.^40^ Because AML cells are exposed to insulin in vivo, we tested the effects of pathway inhibitors in the presence of this agonist.

Our studies demonstrated that insulin altered the phosphorylation of at least certain PI3K-Akt-mTOR mediators for all patients, but even after insulin exposure, the subclassification of patients reflected the constitutive (i.e., preinsulin) phosphorylation profile. Even though the relative effect of insulin (i.e., the percent increase in the MFI for various substrates (Fig. 1) was strongest for patients showing low constitutive activation (Supplementary Fig. 1), patients with the strongest constitutive (preinsulin) activation profile still showed the strongest pathway activation (Supplementary Fig. 3). Thus, even after insulin exposure, the differences in constitutive activation influence patient heterogeneity/clustering.

Our studies of global gene expression suggest that a relatively strong and purely agonistic response (i.e., increased phosphorylation for all tested mediators) was associated with differences in several cell surface markers, especially HLA class II molecules, defensins (important for neutrophils), and erythrocyte markers (especially hemoglobin components). HLA class II molecules are primarily expressed by immunocompetent cells and may thus be regarded as differentiation markers. Taken together, these observations suggest that insulin responsiveness is associated with cellular differentiation status.

We investigated differences in global gene expression as well as proteomic and phosphoprotein profiles between patients who showed a general increase in pathway activation in response to insulin and patients showing a divergent response. Differences in insulin responsiveness were then associated with differences in the regulation of differentiation, transcription, protein modulation, and metabolism. These differences can thus be used to identify distinct patient subsets with regard to insulin responsiveness (Fig. 1). Future clinical studies should therefore investigate whether patient subsets identified by mRNA/proteomic profiling also differ in their in vivo sensitivity to therapy, especially pathway inhibitors.

Our proteomic and phosphoproteomic studies suggested that the patient subset characterized by general PI3K-Akt-mTOR activation in response to insulin differed from other patients with regard to metabolic regulation. Targeting cell metabolism is now considered a possible therapeutic strategy in AML;^29^ therefore, we investigated whether these patients differed in their susceptibility to metabolic inhibition. Glycolysis is an important part of the energy metabolism in malignant cells,^29^ and differences in glycolysis appear to be important for in vivo chemosensitivity in AML.^28^ We investigated the antiproliferative effects of three inhibitors directed against molecular targets that are relevant to glycolysis: (i) hexokinase, which is the rate-limiting enzyme in glycolysis; (ii) the lactate transporter Monocarboxylate Transporter 1 (MCT-1); and (iii) the rate-limiting enzyme in the pentose phosphate pathway (a parallel pathway to glycolysis), glucose-6-phosphate dehydrogenase (G6PD). Hexokinase inhibition had a weaker antiproliferative effect in patients who showed a strong and uniform response to insulin exposure than in other patients.

Akt is an important regulator of glycolysis and oxidative phosphorylation in cancer cells. These effects are mediated by multiple mechanisms, including hexokinase expression.^13^ Furthermore, there is a functional link between Akt-mTOR activation and MCT-1 expression^41^ and possibly between Akt and the pentose phosphate pathway.^42^ For these reasons, we performed an additional clustering analysis to subclassify our patients based on the insulin-induced phosphorylation of Akt and its immediate downstream mediator mTOR. This analysis identified two main patient subsets, and the cluster showing strong insulin-induced phosphorylation was associated with significantly weakened antiproliferative effects of all three metabolic inhibitors. Taken together, our results show that stronger activation of Akt-TOR in response to insulin is associated with weaker antiproliferative effects of metabolic inhibitors, and the subset of patients with both Akt-mTOR activation and strong general PI3K-Akt-mTOR pathway activation are especially resistant to inhibition of hexokinase, the rate-limiting enzyme of glycolysis.

We investigated the effects of three pathway inhibitors on the phosphorylation profile of AML patient cells in the presence of insulin. The phosphorylation of the targeted molecules was generally decreased, the corresponding upstream mediators usually showed unaltered or increased phosphorylation, and the negative feedback effect of mTOR on Akt phosphorylation was maintained.^43^ Each inhibitor had similar enhancing or inhibitory effects for a large majority of patients only for certain mediators (see Fig. 5), whereas they had divergent effects for the other mediators. These general effects differed between the inhibitors; PI3K and Akt inhibition had similar effects, whereas mTOR inhibition differed. Thus, despite the extensive heterogeneity among AML patients, each inhibitor shows similar, and thereby predictable, effects on some of the mediators in all or almost all patients.

Our experimental studies show that patients are heterogeneous with regard to the constitutive AML cell activation of the PI3K-Akt-mTOR pathway; this heterogeneity is increased by insulin and maintained in the presence of pathway inhibitors. Patients can be subclassified based on the effects of insulin on the phosphorylation of pathway mediators, and these subsets differ in their global gene expression as well as their proteomic and phosphoproteomic profiles. The subset with the strongest PI3K-Akt-mTOR pathway activation in response to insulin also shows weakened antileukemic effects of metabolic inhibitors. These heterogeneities may merit consideration when results from clinical studies of PI3K-Akt-mTOR or metabolic targeting are being examined. Our results may thus contribute to a scientific basis for the identification of patient subsets that are particularly susceptible to these antileukemic strategies.

## Materials and methods

### AML patients

The study was approved by the Regional Ethics Committee (REK III 060.02, REK Vest 2013/634, REK Vest 2015/1410) and samples collected after written informed consent. The clinical and biological characteristics of the 76 consecutive (i.e., unselected) patients included in the study are summarized in Table 3. All patients had a high number and/or percentage of peripheral blood AML blasts; leukemic peripheral blood mononuclear cells could therefore be isolated by density gradient separation alone (Lymphoprep, Axis-Shield, Oslo, Norway) and generally contained at least 95% leukemic blasts. The contaminating cells were mainly small lymphocytes. These enriched AML cells were cryopreserved and stored in liquid nitrogen; the cryopreservation solution was insulin-free RPMI 1640 (Sigma-Aldrich, St. Louis, Missouri) medium supplemented with 10% dimethylsulfoxide and 20% inactivated fetal calf serum (final concentrations).

### Analysis of PI3K-Akt-mTOR activation

As described previously, flow cytometry was used to examine the phosphorylation of selected mediators in the main track of the PI3K-Akt-mTOR network.^4^ A detailed methodological description is included in the Supplementary Information. Finally, previous studies have shown that our cryopreserved AML cell populations usually have a viability of 70–75% and a purity of at least 90%;^15^ this was also seen from the present analyses (live-dead gating; scatter differences).

For pharmacological studies, AML cells were incubated with human insulin 10 µg/mL (Sigma-Aldrich, St. Louis, MO, USA), the PI3K class I inhibitor GDC0941 (Axon Medchem BV, Groningen, The Netherlands), the Akt inhibitor MK2206 (Selleckchem, Houston, TX, USA) or the mTOR inhibitor rapamycin (LC Laboratories, Woburn, MA, USA). AML cells were incubated for 15 minutes with insulin or in medium alone before analysis. For the investigation of pharmacological effects, the cells were incubated with or without insulin for 15 minutes prior to an additional 15-min incubation with a pharmacological agent, followed by flow cytometric analysis. All inhibitors were tested at a final concentration (100 nM) that showed antiproliferative effects in a ^3^H-thymidine incorporation assay.^44^

### Analysis of global gene expression profiles

Our methods for analysis of global gene expression profiles have been described previously;^45^ a more detailed description is given in the Supplementary Information.

### Proteomic analysis

The methods for analysis of proteomic profiles in primary human AML cells have been described in detail previously.^46–48^ Briefly, each patient sample was spiked with an AML-derived super-SILAC mix and prepared according to a filter-aided sample preparation protocol,^49^ followed by liquid chromatography coupled with a Q Exactive HF hybrid quadrupole-Orbitrap mass spectrometer. Data were analyzed as described previously.^5^

### Analysis of cytokine-dependent AML cell proliferation

AML cell proliferation was analyzed in a 7-day ^3^H-thymidine incorporation assay as described in detail previously.^6^ Pharmacological effects on AML cell proliferation are presented as proliferation in drug-containing cultures relative to proliferation in drug-free controls. We tested the effects of three inhibitors that can affect the glycolytic pathway: (i) the hexokinase inhibitor lonidamine (final concentration 300 μM; Sigma-Aldrich, St. Louis, MO, USA); (ii) the MCT-1 inhibitor AZD3965 (200 nM; Cayman Chemicals, Ann Arbor, MI, USA); and (iii) 6-AN (50 μM; Cayman Chemical), an inhibitor of the pentose phosphate pathway. The selected concentrations were based on initial dose-response experiments (data not shown).

### Data collection, bioinformatical, and statistical analyses

Bioinformatic analyses were performed using the software J-Express 2012 (MolMine AS, Bergen, Norway). For hierarchical clustering analysis, all values were calculated using fold change on the inverse hyperbolic sine (arcsine) scale, with median values for each target group as controls. Complete linkage and Pearson correlation were used as the linkage method and distance measurement, respectively. Statistical analyses were performed using the IBM Statistical Package for the Social Sciences (SPSS) version 23 (Chicago, IL, USA). The Mann–Whitney *U*-test was used to compare different groups, the chi-squared test or Fischer’s exact test for analysis of categorized data and Kendall’s tau-b test for correlation analyses. *P-*values < 0.05 were regarded as statistically significant.

## Supplementary information

Supplementary information Effects of insulin and pathway inhibitors on PI3K-AKT-mTOR phosphorylation profile in acute myeloid leukemia cells -

## Data Availability

The datasets generated during and/or analysed during the current study are available from the corresponding author on reasonable request. The collected data are stored in anonymous form.
