# Supplementary material for: Effects of insulin and pathway inhibitors on the PI3K-Akt-mTOR phosphorylation profile in acute myeloid leukemia cells
Source: Signal Transduct Target Ther. 2019 Jun 19;4:20. doi: 10.1038/s41392-019-0050-0 (PMC6582141; doi:10.1038/s41392-019-0050-0)
Supplement: Supplementary file 1 — Supplementary information Effects of insulin and pathway inhibitors on PI3K-AKT-mTOR phosphorylation profile in acute myeloid leukemia cells - [file 41392_2019_50_MOESM1_ESM.docx]

## SUPPLEMENTARY INFORMATION

**EFFECTS OF INSULIN AND PATHWAY INHIBITORS ON PI3K-AKT-mTOR PHOSPHORYLATION PROFILE IN ACUTE MYELOID LEUKEMIA CELLS**

Ina Nepstad^1^, [ina.nepstad@uib.no](mailto:ina.nepstad@uib.no)

Kimberley J. Hatfield ^1,2^, [Kimberley.Hatfield@uib.no](mailto:Kimberley.Hatfield@uib.no)

Ida Sofie Grønningsæter^1^, [Ida.Gronningseter@uib.no](mailto:Ida.Gronningseter@uib.no)

Elise Aasebø^4^, [Elise.Aasebo@uib.no](mailto:Elise.Aasebo@uib.no)

Maria Hernandez-Valladares^4^, [Maria.Hernandez-Valladares@uib.no](mailto:Maria.Hernandez-Valladares@uib.no)

Karen Marie Hagen^1^, [Marie.Hagen@uib.no](mailto:Marie.Hagen@uib.no)

Kristin Paulsen Rye^1^, [Kristin.Rye@uib.no](mailto:Kristin.Rye@uib.no)

Frode Berven^4^, [Frode.Berven@uib.no](mailto:Frode.Berven@uib.no)

Frode Selheim^4^, [Frode.Selheim@uib.no](mailto:Frode.Selheim@uib.no)

Håkon Reikvam^1,3^, [Hakon.Reikvam@uib.no](mailto:Hakon.Reikvam@uib.no)

Øystein Bruserud^1,3^, [oystein.bruserud@helse-bergen.no](mailto:oystein.bruserud@helse-bergen.no)

^1^ Section for Hematology, Department of Clinical Science, University of Bergen, Norway.

^2^ Department of Immunology and Transfusion Medicine, Haukeland University Hospital, Bergen, Norway.

^3^ Section for Hematology, Department of Medicine, Haukeland University Hospital, Bergen, Norway.

^4^ Department of Biomedicine, Faculty of Medicine and Dentistry, University of Bergen, Jonas Lies vei 91, 5009 Bergen, Norway

Corresponding author: Øystein Bruserud, Section for Hematology, Department of Medicine, Haukeland University Hospital, N-5021 Bergen, Norway.

Phone: 0047 928 98 765. Fax 0047 55972950. E-mail: [oystein.bruserud@helse-bergen.no](mailto:oystein.bruserud@helse-bergen.no)

**DETAILED METHODOLOGICAL DESCRIPTIONS**

**Flow cytometric analysis of PI3K-Akt-mTOR activation**

Flow cytometry was used to examine the phosphorylation of selected mediators in the main track of the PI3K-Akt-mTOR network [PMID 29382066]. Briefly, primary AML cells were incubated for 20 minutes in RPMI-1640 (Sigma-Aldrich), fixed in 1.5% paraformaldehyde (PFA), permeabilized by 100% methanol, and stored at -80°C until used. The cells were then rehydrated using 2 ml phosphate-buffered saline (PBS), resuspended, and centrifuged before they were washed twice with PBS. Cells were thereafter resuspended in 150 μl PBS with 0.1% BSA (Sigma, St. Louis, Missouri) and blocked by immunoglobulin (Octagam; Octapharma, Jessheim, Norway) and 1% BSA. The cells were finally distributed onto 96-well plates (1 x 10^5^ cells per sample) and stained. All staining panels included the same live/dead discriminator, either FITC or Alexa Fluor^®^ 647 Mouse anti-Cleaved PARP (Asp214). Three blank samples were also included on each plate. Three directly conjugated dyes were used: (i) Alexa Fluor® 647 for PDK1 pS241, PKCα pT497, Akt pS473, 4EBP1 pT36/pT45, elF4E pS209 and S6 pS244; (ii) phycoerythrin (PE) for Akt pT308, mTOR pS2448, and S6 pS240; and (iii) V450 for S6 pS235/pS236 (all antibodies from BD Pharmingen, Franklin Lakes, NJ, US). Viable leukemic cells were identified and analyzed by flow cytometry based on live/dead staining, doublet discrimination, CD45 staining, and forward/side scatter. Dead cells, doublets, and contaminating lymphocytes were excluded from later analyses. Flow cytometric analysis was acquired on a BD FACS Verse 8-color cytometer (BD Biosciences) and data analysis was performed using FlowJo 10.0.7 software (Tree Star, Inc., Ashland, OR, USA).

**Analysis of global gene expression profiles**

RNA isolation was performed according to QIAgen’s instructions for the RNeasy with the DNase treatment protocol for the QIAcube (QIAGEN, Hilden, Germany). All microarray analyses were performed using the Illumina iScan Reader and based on fluorescence detection of biotin-labeled cRNA. For each sample, 300 ng total RNA was reversely transcribed, amplified, and Biotin-16-UTP-labeled using the Illumina TotalPrep RNA Amplification Kit (Applied Biosystems/Ambion; Foster City, CA, USA). The amount and quality of the biotin-labeled cRNA was controlled by the NanoDrop spectrophotometer and the Agilent 2100 Bioanalyzer before 750 ng of biotin-labeled cRNA was hybridized to the HumanHT-12 V4 Expression BeadChip that targets 47 231 probes (Release 38). The data from the array scan (IlluminaiScan Reader) were examined in GenomeStudio and J-Express 2012 (MolMine AS, Bergen, Norway) for quality control [PMID 28877686]. Quantile normalization of arrays were performed prior to the data being compiled into an expression profile data matrix. Genes were merged using the max probe for merge statistics prior to analysis. GO terms were identified using the Gene Ontology website (<http://www.geneontology.org/GO.current.annotations.shtm>).

**Analysis of cytokine-dependent AML cell proliferation**

AML cell proliferation was analyzed in a 7 days ^3^H-thymidine incorporation assay as described in detail previously [PMID 17341267 and 24383842]. Briefly, enriched human AML cells were cultured in microtiter well (50 x 10^4^ cells per well, 200 μl medium/well) and incubated in a humidified atmosphere of 5% CO_2_; ^3^H-thymidine was added after six days and nuclear incorporation was assayed on day 7. Cells were cultured in the Stem Span SFEM medium (Stem Cell Technologies, Vancouver, Canada) supplemented with Flt3 ligand, granulocyte-macrophage colony-stimulating factor and Stem cell factor (all from Peprotech, Ricky Hill NJ, USA; final concentrations 20 pg/ml); the Stem Span medium is supplemented with insulin (manufacturer’s information). All calculations are based on the median ^3^H-thymidine incorporation (counts per minute, cpm) of triplicate cultures. Pharmacological effects on AML cell proliferation are presented as the proliferation in drug-containing cultures relative to the proliferation in drug-free controls. We tested the effects of three inhibitors that are relevant for glycolysis: (i) Lonidamine (final concentration 300 μM; Sigma-Aldrich, St. Louis, MO, USA) is a hexokinase inhibitor, the rate-limiting enzyme in glycolysis; (ii) AZD3965 (200 nM; Cayman Chemicals, Ann Arbour, MI, USA) inhibits Monocarboxylate Transporter 1 (MCT-1), a transporter of lactate across membranes; and (iii) 6-amino-nicotinamide (50 μM; Cayman Chemical) inhibits glucose-6-phosphate dehydrogenase (G6PD) and 6-phosphogluconate dehydrogenase (6PGD); G6PD being the rate-limiting enzyme in the pentose phosphate pathway that is a parallel pathway to glycolysis. The selected concentrations were based on initial dose-response experimens (data not shown).

**
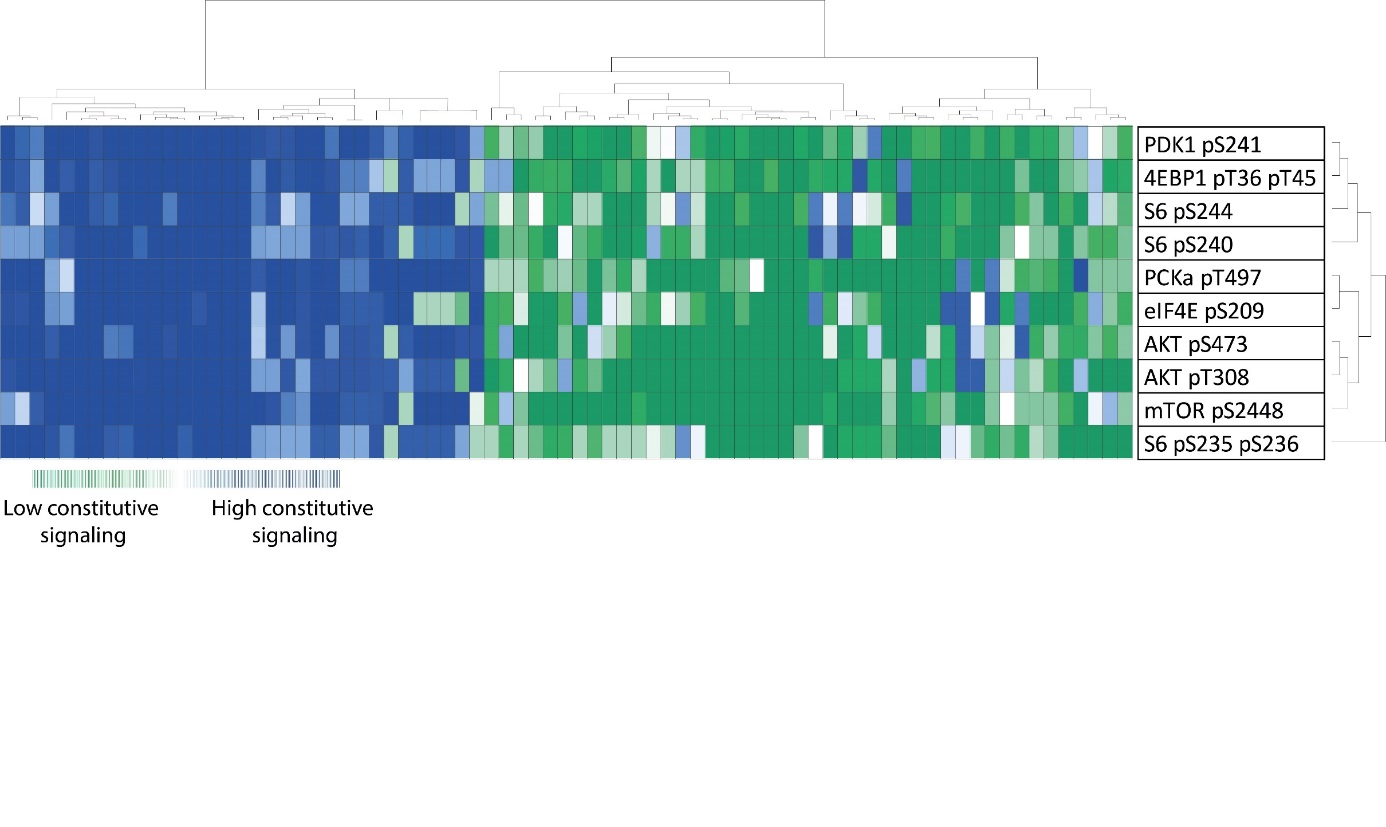
**

**Supplementary Figure 1. Clustering analysis of the constitutive PI3K-Akt-mTOR signaling profile for enriched primary AML cells.** The 76 consecutive patients could be divided into to main clusters/subsets based on this analysis; the right subset of patients showed generally strong constitutive signaling and the left main subset was characterized by generally weaker constitutive pathway activation (green indicates high phosphorylation and blue low phosphorylation).


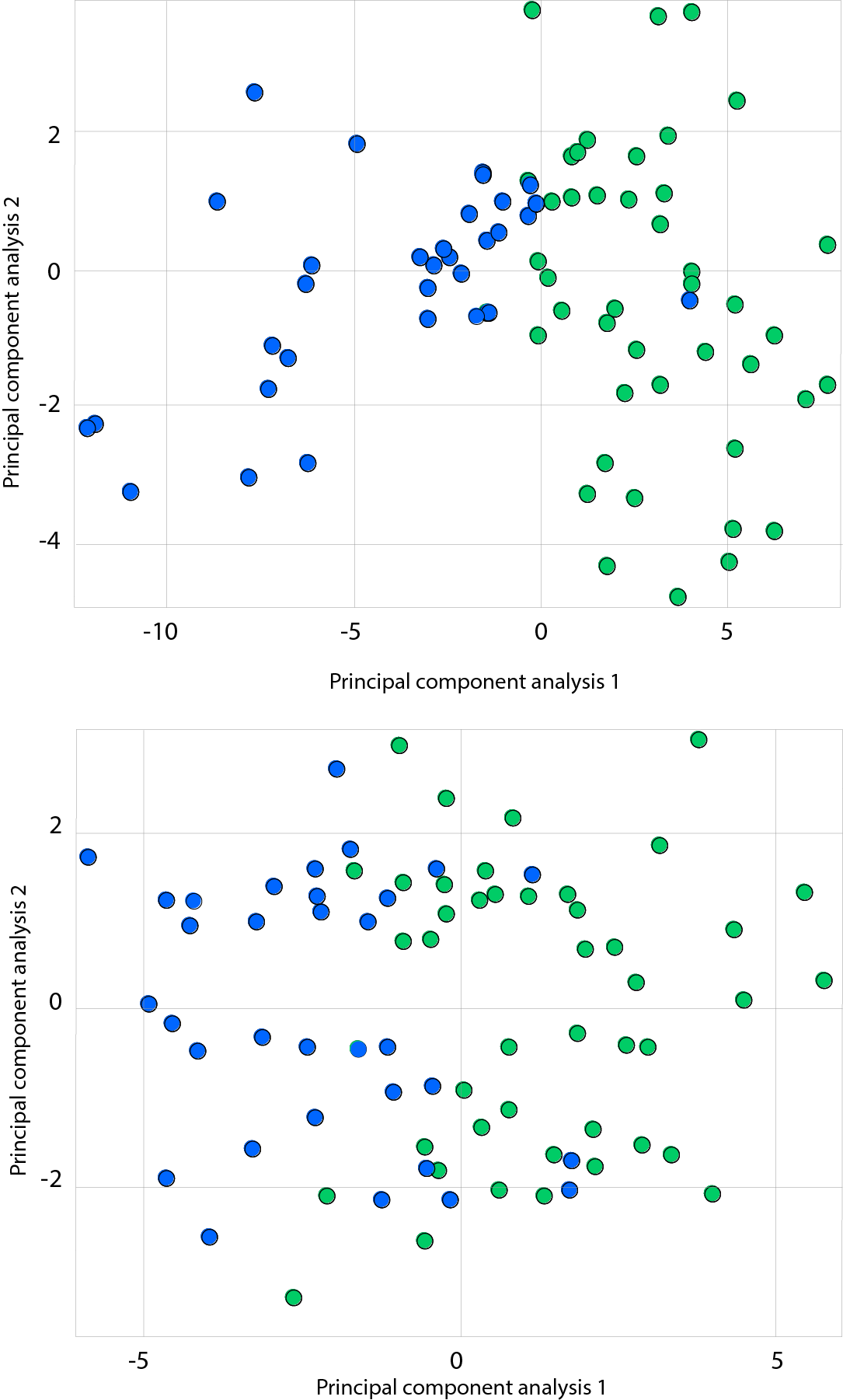


**Supplementary Figure 2.** **Principal component analysis (PCA) comparing the constitutive activation of the PI3K-Akt-mTOR pathway (upper diagram) and the pathway activation after incubation with insulin (lower diagram).** Primary AML cells from all 76 patients were included in both analyses and the patients were separated into two subsets (i.e. the two main clusters) based on the hierarchical clustering analysis presented in Supplementary Figure 1. Blue symbols means that the patients belonged to the lower main cluster in Supplementary Figure 1 and showed relatively low constitutive activation, whereas green symbols indicate patients that showed strong pathway activation in this clustering analysis. The analyses were based on the MFI values for patients incubated in medium alone (upper diagram) and on the relative insulin responses (lower diagram; MFI for insulin-containing cultures relative to the MFI for corresponding control cultures), respectively. The upper diagram confirmed that patients could be separated into two main subsets, and there was a relatively small overlap between these two subsets even after exposure to insulin. Thus, the patient heterogeneity reflected in the constitutive pathway activation is largely maintained also after insulin exposure.

**
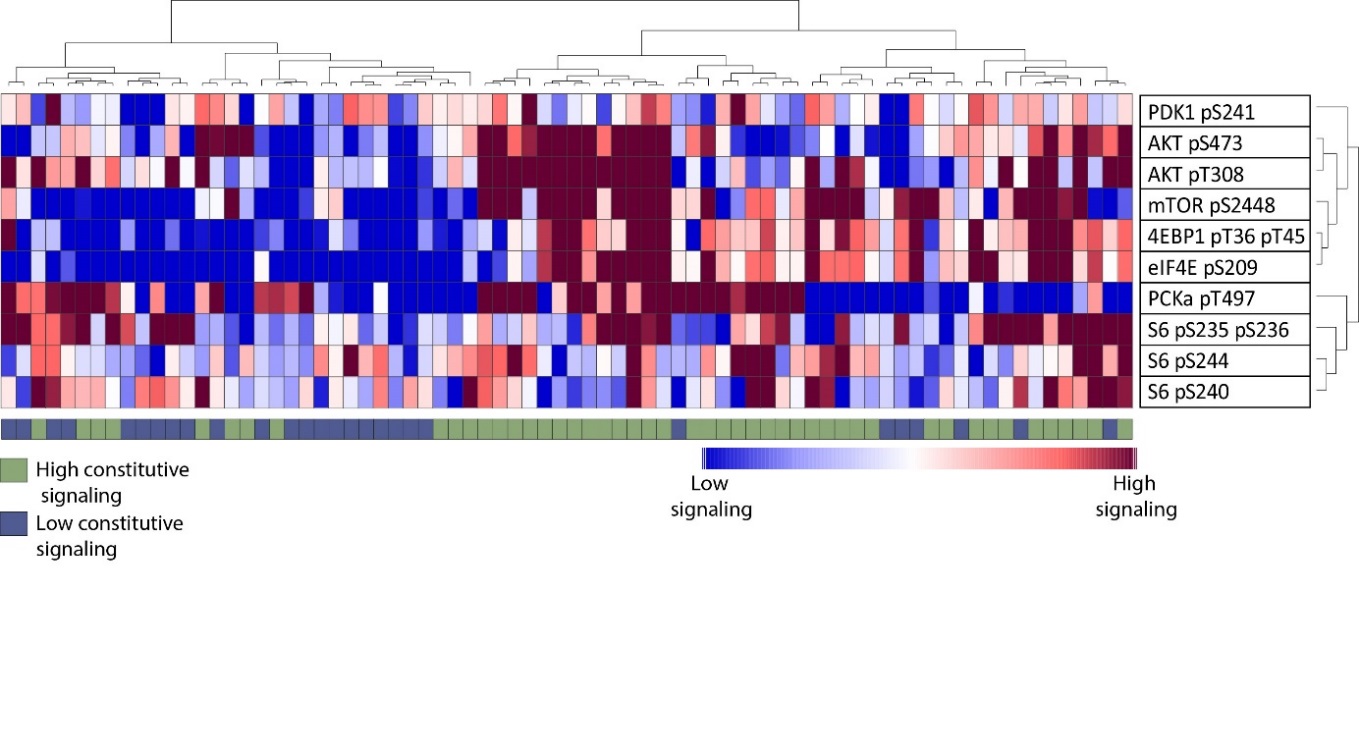
**

**Supplementary Figure 3. Clustering analysis of the PI3K-Akt-mTOR signaling profile for enriched primary AML cells incubated *in vitro* with insulin.** This analysis was based on the absolute phosphorylation level in the presence of insulin, i.e. the MFI values for AML cells after incubation with insulin. The 76 patients could be divided into to main clusters/subsets based on this analysis; one subset of patients showed generally strong signaling (right) and s second subset characterized by generally weaker signaling (left). The column below the cluster indicates that most patients with high constitutive signaling (Supplementary Figure 1) were included in the right cluster. Thus,the patient heterogeneity detected in the analysis in constitutive pathway activation contributes to the patient heterogeneity also after exposure to the agonistic insulin.


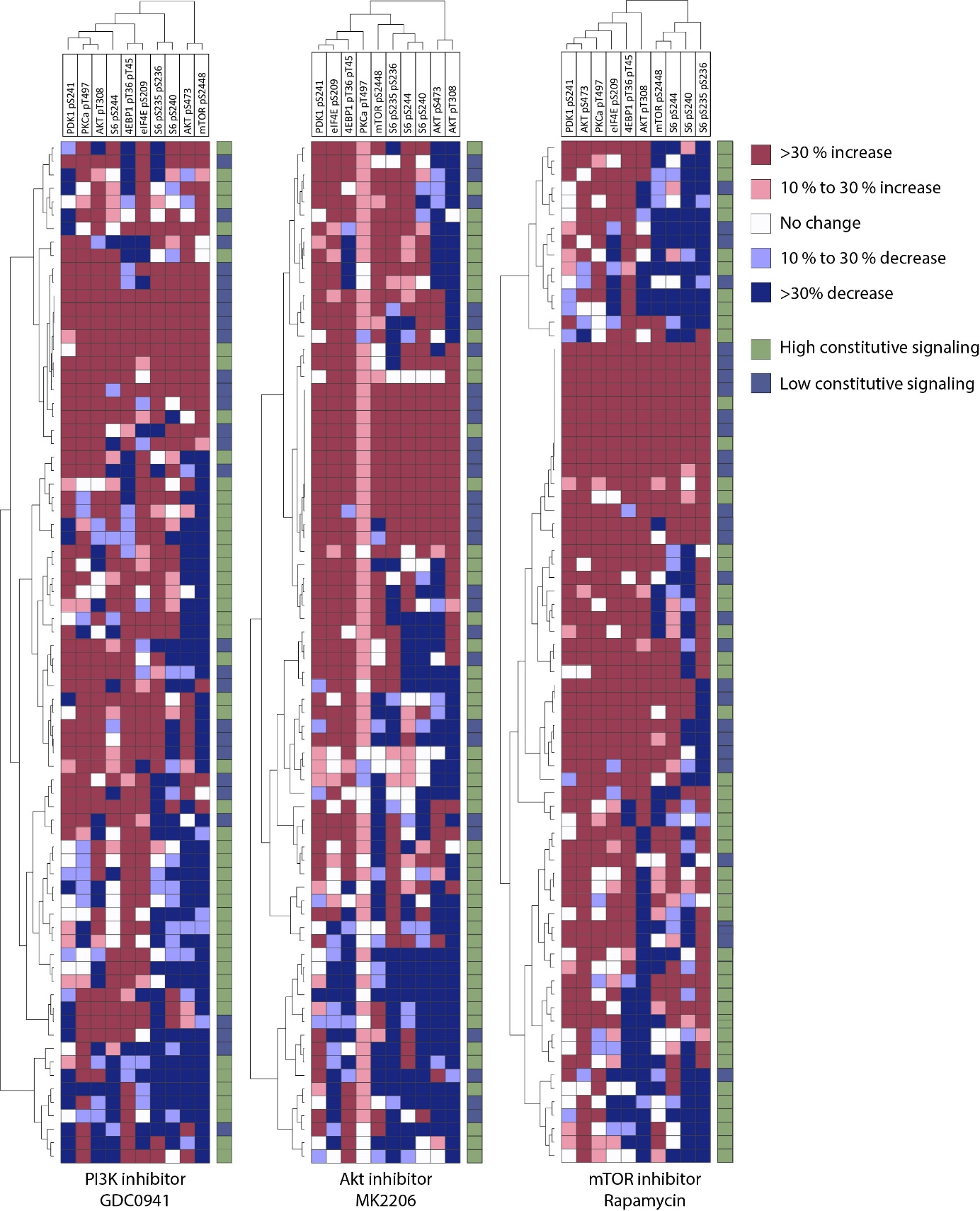


**Supplementary Figure 4. Clustering analysis of the PI3K-Akt-mTOR signaling profile for enriched primary AML cells incubated *in vitro* with insulin+pathway inhibitor.** The leukemic cells were derived from all 76 patients and incubated with insulin alone or plus one of the three pathway inhibitors (see lower part of the figure). The results are presented as the relative MFI, i.e. the percent increcrease/decrease of the MFI for each mediator when cells were incubated with insulin plus inhibitor compared with the corresponding MFI for leukemic cells incubated in medium alone. This means that the red colour indicates that the MFI is still higher than the corresponding constitutive value even in the presence of an inhibitor (i.e. the inhibitor does not completely reverse the insulin effect), whereas the blue colour indicates that the MFI value is lower than the corresponding constitutive MFI (i.e. the insulin effect is more than reversed) value after exposure to an inhibitor.

**Supplementary Table 1. The effect of insulin on the activation status of individual mediators in the PI3K-Akt-mTOR pathway; a summary of the results for primary AML cells derived from 76 patients.** The MFI for AML cells incubated with insulin were compared with the MFI for corresponding cultures prepared in medium alone. The results are presented as the number of patients with at least a 30 % increase of the MFI for the insulin-containing cultures, only a minimal insulin effect and an insulin-induced decrease in the MFI of at least 30 % compared with the corresponding medium controls.

|  | **Increased phosphorylation**  **(>30 % increased MFI)** | **Minimal insulin effect (<30% altered MFI)** | **Decreased phosphorylation**  **(>30 % decreased MFI)** |
| --- | --- | --- | --- |
| PDK1 pS241 | 55 | 21 | - |
| PCKa pT497 | 51 | 25 | - |
| AKT pS473 | 48 | 27 | 1 |
| AKT pT308 | 36 | 37 | 3 |
| mTOR pS2448 | 70 | 6 | - |
| 4EBP1 pT36 pT45 | 67 | 9 | - |
| eIF4E pS209 | 49 | 15 | 12 |
| S6 pS235 pS236 | 55 | 20 | 1 |
| S6 pS244 | 44 | 22 | 10 |
| S6 pS240 | 43 | 21 | 12 |

**Supplementary Table 2. A comparison of global gene expression profiles for patients with relatively strong and weaker insulin responsiveness (Figure 1, cluster I patients versus other patients).** The table describes the differentially expressed genes when comparing primary AML cells showing a strong activation of the PI3K-Akt-mTOR pathway in response to insulin versus patients showing decreased phosphorylation of at least one pathway mediator after insulin exposure. The shaded genes represent genes that were included in several significant GO terms (see Supplementary Table 4)

| **THE HLA CLUSTER – CELL SURFACE MOLECULES** | | |
| --- | --- | --- |
| HLA-DRB1 | *HLA-class II molecules.* The HLA class II molecules are normally expressed mainly on immunocompetent cells, but they can also be expressed by primary human AML cells. | Antigen presentation |
| HLA-DPA1 |  |  |
| HLA-DR1 |  |  |
| HLA-DMB |  |  |
| HLA-DMA |  |  |
| HLA-DQA1 |  |  |
| HLA-DRB3 |  |  |
| CD36 | *CD36.* The encoded protein serves as a receptor for thrombospondins that are widely distributed proteins involved in a variety of adhesive processes. It binds to collagen, thrombospondin, anionic phospholipids, oxidized LDL and long chain fatty acids; it may also function in the transport and/or as a regulator of the transport of fatty acids. | Cell adhesion  Cell metabolism |
| CD74 | *CD74.* The protein associates with class II major histocompatibility complex (MHC) and is an important chaperone that regulates antigen presentation; it also serves as cell surface receptor for the cytokine macrophage migration inhibitory factor (MIF) and thereby initiates survival pathways and cell proliferation. | HLA association  Cytokine receptor |
| MPO | *Myeloperoxydase.* This heme protein is synthesized during myeloid differentiation and constitutes the major component of neutrophil azurophilic granules. This enzyme produces mediators that are central to the microbicidal activity of neutrophils. | Mitochondria |
| LAMA5 | *Laminin alpha 5.* Laminins are a family of extracellular matrix glycoproteins; they are important for cell adhesion, differentiation, migration, and signaling. Laminins are composed of the 3 non identical chains laminin alpha, beta and gamma. The protein encoded by this gene is the alpha-5 subunit of laminin-10 (laminin-511), laminin-11 (laminin-521) and laminin-15 (laminin-523). | Adhesion  Migration |
| KIT | *KIT proto-oncogene receptor tyrosine kinase.* The proto-oncogene c-kit is a type 3 transmembrane receptor for the growth factor Stem Cell Factor that is important in acute myeloid leukemia. (Reikvam H et al., Cell Prolif 2013, 46, 554; Bruserud Ø et al., Haematologica 2003, 88, 416). | Cytokine receptor  Proliferation  Survival |
| SELL | *Selectin L.* This is a cell surface adhesion molecule that belongs to a family of adhesion/homing receptors. | Adhesion  Migration |
| **THE ERYTHROID CLUSTER** | | |
| HBQ1 | *Hemoglobin subunit theta 1.* Theta-globin mRNA is not found in adult erythroid or other non-erythroid tissue. It is a member of the human alpha-globin gene cluster and seems to have a functional role for the peptide in specific cells, possibly those of early erythroid tissue. It can be expressed in breast cancer (Borgan E, Mol cancer 2013, 7:130). | Cancer associated  Erythrocytes |
| EPB49/DMTN | *Dematin actin binding protein.* The protein is an actin binding and bundling protein that can stabilize and attach the spectrin/actin cytoskeleton to the cell membrane in a phosphorylation-dependent manner. Loss of heterozygosity is thought to play a role in prostate cancer progression. | Cytoskeleton  Prostate cancer  Erythrocytes |
| EPB42 | *Erythrocyte membrane protein band 4.2.* This is an ATP-binding protein which may regulate the association of protein 3 with ankyrin. | Ankyrin  Cytoskeleton  Erythrocytes |
| SLCA1  (MCHR1) | *Melanin concentrating hormone receptor 1.* This plasma membrane protein is a member of the G protein-coupled receptor family 1 and binds melanin-concentrating hormone. The encoded protein can inhibit cAMP accumulation and stimulate intracellular calcium flux | GPCR |
| CA1 | *Carbonic anhydrase 1.* Carbonic anhydrases (CAs) are a large family of zinc metalloenzymes that catalyze the reversible hydration of carbon dioxide. They participate in a variety of biological processes, including respiration and acid-base balance. They show extensive diversity in tissue distribution and in their subcellular localization. This cytosolic protein CA1 protein is found at the highest level in erythrocytes. | Acid-base balance  Erythrocytes |
| ALAS2 | *5'-aminolevulinate synthase 2.* The protein is an erythroid-specific mitochondrial enzyme. The encoded protein catalyzes the first step in the heme biosynthetic pathway. | Mitochondria  Erythrocytes |
| GYPC | *Glycophorin C.* Glycophorin C (GYPC) is an integral membrane glycoprotein. It is a minor species carried by human erythrocytes, but plays an important role in regulating the mechanical stability of cells. | Erythrocytes  Cytoskeleton? |
| HBA1 | *Hemoglobin subunit alpha 1.* It is a hemoglobin protein encoded by the *HBA1* gene. Two alpha chains plus two beta chains constitute HbA, which in normal adult life comprises about 97% of the total hemoglobin; alpha chains combine with delta chains to constitute HbA-2 | Erythrocytes |
| ITPR3 | *Inositol 1,4,5-trisphosphate receptor type 3.* This gene encodes a receptor for inositol 1,4,5-trisphosphate, a second messenger that mediates the release of intracellular calcium. The receptor contains a calcium channel. Knockout studies in mice suggest that type 2 and type 3 inositol 1,4,5-trisphosphate receptors play a key role in exocrine secretion underlying energy metabolism and growth. | Secretion  Metabolism  Calcium metabolism |
| HBD | *Hemoglobin subunit delta.* Two alpha chains plus two delta chains constitute HbA-2. | Erythrocytes |
| ICA1 | *Islet cell autoantigen.* This protein is found both in the cytosol and as membrane-bound form on the Golgi complex and immature secretory granules. | Cytosol, Golgi  Secretory granules |
| SERPINE2 | *Serpin family E member 2.* This protein is a member of the serpin family of proteins, a group of proteins that inhibit serine proteases. Thrombin, urokinase, plasmin and trypsin are among the proteases that this family member can inhibit. | Protease inhibitor |
| **INTRACELLULAR SIGNALING, TRANSCRIPTION AND NEUTROPHIL DIFFERENTIATION** | | |
| ARAP3 | *ArfGAP with RhoGAP domain, ankyrin repeat and PH domain 3.* This gene encodes a phosphoinositide binding protein. The ARF-GAP and RHO-GAP domains cooperate in mediating rearrangements in the cell cytoskeleton and cell shape. It is a specific PtdIns(3,4,5)P3/PtdIns(3,4)P2-stimulated Arf6-GAP protein. | Cytoskeleton |
| BCL6 | *B cell CLL/lymphoma 6.* The protein is a zinc finger transcription factor that acts as a sequence-specific repressor of transcription. It can interact with a variety of POZ-containing proteins that function as transcription corepressors. It can be frequently translocated and hypermutated in diffuse large-cell lymphoma (DLCL). | Transcription  Lymphoma |
| S100A12 | *S100 calcium binding protein A12.* The protein is a member of the S100 family of proteins containing 2 EF-hand calcium-binding motifs. S100 proteins are localized in the cytoplasm and/or nucleus and involved in the regulation of a number of cellular processes such as cell cycle progression and differentiation. This protein is proposed to be involved in specific calcium-dependent signal transduction pathways and have regulatory effect on cytoskeletal components | Calcium metabolism  Signal transduction  Cytoskeleton |
| IRS2 | *Insulin receptor substrate 2.* The insulin receptor substrate 2 is a cytoplasmic signaling molecule that mediates effects of insulin, insulin-like growth factor 1, and other cytokines by acting as a molecular adaptor between diverse receptor tyrosine kinases and downstream effectors. It gene is phosphorylated by the insulin receptor tyrosine kinase upon receptor stimulation, as well as by an interleukin 4 receptor-associated kinase. | Intracellular signaling  Insulin |
| HP | *Haptoglobin.* This gene encodes a preproprotein, which is processed to yield both alpha and beta chains, which subsequently combine as a tetramer to produce haptoglobin. Haptoglobin functions to bind free plasma hemoglobin | Heme binding |
| CXCR4 | *C-X-C motif chemokine receptor 4.* This CXC chemokine receptor is specific for stromal cell-derived factor-1 or CXCL12. This receptor is important for AML cell proliferation and may also represent a therapeutic target in human AML (Peled A, Tavor S, Theranostics 2013, 3: 34-9). | AML proliferation  Chemokine |
| MT1A | *Metallothionein 1A.* The metallothionein family of genes encodes low molecular weight proteins that bind divalent heavy metal ions that act as anti-oxidants and protect against hydroxyl free radicals. | Oxidative stress |
| MT1E | *Metallothionein 1E.* Very low expression in normal bone marrow. | Oxidative stress? |
| ID2 | *Inhibitor of DNA binding 2.* The protein belongs to the inhibitor of DNA binding family, members of which are transcriptional regulators that inhibit the functions of basic helix-loop-helix transcription factors in a dominant-negative manner by suppressing their heterodimerization partners | Transcription |
| LAMC1 | *Laminin subunit gamma 1.* Laminins are a family of extracellular matrix glycoproteins. They have been implicated in a wide variety of biological processes including cell adhesion, migration and signaling. Laminins are composed of 3 non identical chains: laminin alpha, beta and gamma This gene encodes the gamma chain isoform laminin, gamma 1. | Adhesion  Migration Extracellular matrix |
| KLF9 | *Kruppel like factor 9.* This protein is a transcription factor that binds to GC box elements located in the promoter. Binding of the encoded protein to a single GC box inhibits mRNA expression while binding to tandemly repeated GC box elements activates transcription. | Transcription |
| DEFA1 | *Defensin alpha 1.* Defensins are a family of antimicrobial and cytotoxic peptides that are abundant in the granules of neutrophils Members of the defensin family are highly similar in protein sequence and distinguished by a conserved cysteine motif. This encoded defensing likely plays a role in phagocytosis. | Defensin-neutrophils  Phagocytosis |
| DEFA3 | *Defensin alpha 3.* This defensing is found in the microbicidal granules of neutrophils and likely plays a role in phagocyte-mediated host defense. | Defensin-neutrophils  Phagocytosis |
| DEFA1B | *Defensin alpha 1B.* This defensing is also found in the microbicidal granules of neutrophils and likely plays a role in phagocyte-mediated host defense. | Defensin-neutrophils  Phagocytosis |
| DEFA4 | *Defensin alpha 4.* This defensin differs from other genes of this family by an extra 83-base segment that is apparently the result of a recent duplication within the coding region. The protein encoded by this gene, defensin, alpha 4, is found in the neutrophils. | Defensin-neutrophils  Phagocytosis |
| MMP9 | *Matrix metalloprotease 9.* Proteins of the matrix metalloproteinase (MMP) family are involved in the breakdown of extracellular matrix in normal physiological processes. Most MMP's are secreted as inactive proproteins which are activated when cleaved by extracellular proteinases. MMPs can also contribute to extracellular chemokine activation (Hatfield KJ et al, Curr Med Chem 2010, 17, 4448). This enzyme degrades type IV and V collagens; animal studies suggest that it is also involved in IL-8-induced migration of hematopoietic cells. | Matrix degradation  Chemokine  IL8/CXCL8 |
| DDIT4 | *DNA damage inducible transcript 4.* Relatively high expression in normal bone marrow. This is a stress protein that seems to be associated with prognosis in many human malignancies; in AML it seems to be associated with an adverse prognosis. It also seems important for the chemosensitivity (i.e. susceptibility to steroids) of human acute lymphoblastic leukemia cells (Pinto JA et a., Sci Rep 2017, 7, 1526; Benyoucef A et al., Stem Cells 2015, 33, 2268; Wolff NC et al., Mol Cancer Res 2014, 12, 67). | Stress response  Chemosensitivity |
| **MISCELLANEOUS** | | |
| SPP1/SSP6 | *SUMO specific peptidase 6.* Ubiquitin-like molecules, such as SUMO1, are structurally related to ubiquitin and can be ligated to target proteins in a similar manner as ubiquitin. However, covalent attachment of UBLs does not result in degradation of the modified proteins. SUMO1 modification is implicated in the targeting of RANGAP1 to the nuclear pore complex, as well as in stabilization of I-kappa-B-alpha (NFKBIA) from degradation by the 26S proteasome. SENPs also display isopeptidase activity for deconjugation of SUMO-conjugated substrates | Posttranscriptional modification |
| IL8/CXCL8 | *Interleukin 8.* The encoded chemokine is constitutively released at high levels by primary human AML cells (Bruserud Ø et al., Haematologica, 2007, 92, 332). This chemokine is also a potent angiogenic factor. | Cell communication |
| FOXC1 | *Forkhead box C1.* This is a member of the forkhead family of transcription factors. The specific function of this gene has not yet been determined. | Transcription |

**Supplementary Table 3. A summary of the genes described in more detail in Supplementary Table 2.** Differentially expressed genes when comparing primary human AML cells with a strong response to insulin (Figure 1, cluster I) and AML cells showing decreased substrate phosphorylation for at least one substrate in the PI3K-Akt-mTOR pathway (Figure 1, clusters II-IV).

| *Differentiation markers (n=11):* | |
| --- | --- |
|  | Erythroid: HBQ1, EPB49, EPB42, ALAS2, GYPC, HBA1, HBD  Neutrophil/phagocytosis: DEFA1, DEFA3, DEFA1B, DEFA4 |
| *Cell surface molecules, adhesion molecules, matrix molecules (n=13):* | |
|  | HLA molecules: HLADRB1, HLADPA1, HLADR1, HLADMB, HLADQA1, HLADRB1, CD74 (HLA associated).  Adhesion molecules: CD36, LAMA5, SELL, LLAMC1  Matrix molecules: LAMC1, MMP9 (matrix protease) |
| *Soluble mediators, receptors, downstream intracellular signaling (n=10):* | |
|  | Substrates: IL8/CXCL8, MMP9, SERPINEE1  Receptors: KIT, SLCA1, IRS, CXCR4  Intracellular signaling: S100A12  Posttranscriptional modification of proteins: SSP4  Intracellular secretion/transport: ITPR3 |
| *Mitochondrial function, cell metabolism, oxidative stress (n=8):* | |
|  | Mitochondria: MPO, ALAS2, MT1A, MT1E  Oxidative stress: MT1A, MT1E  Cell metabolism: CD36, ITPR3 (associated with the insulin receptor) |
| *Electrolyte regulation (n=2):* | |
|  | ITPR3, S100A12 |
| *Cytoskeleton (n=5):* | |
|  | EPB49, EPB42, GYPC, ARAP3, S100A12 |
| *Transcriptional regulation (n=4):* | |
|  | BCL6, ID2, KLF9, FOXC1 |
| *Miscellaneous (n=1):* | |
|  | HP |
| *Relevance to malignant diseases (n=7):* | |
|  | Chemosensitivity: PDT4  Increased in cancer: HBQ1, EPB49, BCL6  Growth regulators for primary human AML cells: KIT, CXCR4, IL8/CXCL8 |

Supplementary Table 4. Significantly differing GO terms based on the analysis of 45 differentially expressed genes when comparing strong insulin responders (Figure 1, cluster I) versus other patients (Figure 1, clusters II-IV). The table presents the GO terms, number differentially expressed genes included in the term (count), the percentage of these genes out of all genes belonging to this term (%), the p-value (P-Value) and the corrected p-value (Benjamini). The genes were analyzed with regard to Cellular function, Molecular Function and Biological processes.

| **CELLULAR COMPONENTS** | | | | |
| --- | --- | --- | --- | --- |
| Term | Count | % | P-Value | Benjamini |
| MHC class II protein complex GO:0042613 | 8 | 18,6 | 2,80E-14 | 2,70E-12 |
| endocytic vesicle membrane GO:0030666 | 7 | 16,3 | 7,30E-09 | 2,30E-07 |
| integral component of lumenal side of endoplasmic reticulum membrane GO:0071556 | 6 | 14 | 5,10E-09 | 2,40E-07 |
| transport vesicle membrane GO:0030658 | 6 | 14 | 2,10E-08 | 5,00E-07 |
| clathrin-coated endocytic vesicle membrane GO:0030669 | 6 | 14 | 3,20E-08 | 5,90E-07 |
| ER to Golgi transport vesicle membrane GO:0012507 | 6 | 14 | 1,10E-07 | 1,70E-06 |
| trans-Golgi network membrane GO:0032588 | 6 | 14 | 1,10E-06 | 1,50E-05 |
| lysosomal membrane GO:0005765 | 8 | 18,6 | 2,30E-06 | 2,70E-05 |
| azurophil granule lumen GO:0035578 | 3 | 7 | 3,00E-05 | 3,10E-04 |
| late endosome membrane GO:0031902 | 5 | 11,6 | 7,70E-05 | 7,20E-04 |
| extracellular space GO:0005615 | 12 | 27,9 | 1,40E-04 | 1,20E-03 |
| hemoglobin complex GO:0005833 | 3 | 7 | 3,20E-04 | 2,50E-03 |
| extracellular exosome GO:0070062 | 16 | 37,2 | 7,50E-04 | 5,40E-03 |
| cortical cytoskeleton GO:0030863 | 3 | 7 | 1,10E-03 | 7,40E-03 |
| external side of plasma membrane GO:0009897 | 5 | 11,6 | 1,30E-03 | 7,70E-03 |
| Golgi lumen GO:0005796 | 4 | 9,3 | 1,30E-03 | 8,20E-03 |
| Golgi membrane GO:0000139 | 7 | 16,3 | 1,90E-03 | 1,10E-02 |
| extracellular region GO:0005576 | 11 | 25,6 | 2,50E-03 | 1,30E-02 |
| integral component of plasma membrane GO:0005887 | 10 | 23,3 | 3,60E-03 | 1,80E-02 |
| blood microparticle GO:0072562 | 4 | 9,3 | 4,80E-03 | 2,20E-02 |
| plasma membrane GO:0005886 | 18 | 41,9 | 5,40E-03 | 2,40E-02 |
| laminin-11 complex GO:0043260 | 2 | 4,7 | 6,70E-03 | 2,80E-02 |
| laminin-10 complex GO:0043259 | 2 | 4,7 | 6,70E-03 | 2,80E-02 |
| cell surface GO:0009986 | 6 | 14 | 7,10E-03 | 2,90E-02 |
| haptoglobin-hemoglobin complex GO:0031838 | 2 | 4,7 | 9,00E-03 | 3,50E-02 |
| **MOLECULAR FUNCTION** | | | | |
| Term | Count | % | P-Value | Benjamini |
| MHC class II receptor activity GO:0032395 | 6 | 14 | 1,10E-10 | 1,30E-08 |
| peptide antigen binding GO:0042605 | 5 | 11,6 | 3,90E-07 | 2,30E-05 |
| MHC class II protein complex binding GO:0023026 | 4 | 9,3 | 5,30E-06 | 2,10E-04 |
| oxygen transporter activity GO:0005344 | 3 | 7 | 4,20E-04 | 1,20E-02 |
| cytokine binding GO:0019955 | 3 | 7 | 7,80E-04 | 1,80E-02 |

| **BIOLOGICAL PROCESSES** | | | | | |
| --- | --- | --- | --- | --- | --- |
| Term | Count | % | P-Value |  | Benjamini |
| antigen processing and presentation of peptide or polysaccharide antigen via MHC class II GO:0002495 | 7 | 16,3 | 1,50E-12 | 1,00E+00 | 7,30E-10 |
| antigen processing and presentation of exogenous peptide antigen via MHC class II GO:0019886 | 8 | 18,6 | 1,90E-09 | 1,00E+00 | 4,60E-07 |
| antigen processing and presentation GO:0019882 | 7 | 16,3 | 3,30E-09 | 1,00E+00 | 5,30E-07 |
| defense response to fungus GO:0050832 | 6 | 14 | 4,60E-09 | 1,00E+00 | 5,60E-07 |
| killing of cells of other organism GO:0031640 | 5 | 11,6 | 2,70E-08 | 1,00E+00 | 2,60E-06 |
| immune response GO:0006955 | 11 | 25,6 | 3,80E-08 | 1,00E+00 | 3,10E-06 |
| peptide antigen assembly with MHC class II protein complex GO:0002503 | 4 | 9,3 | 1,20E-07 | 1,00E+00 | 8,70E-06 |
| interferon-gamma-mediated signaling pathway GO:0060333 | 5 | 11,6 | 2,40E-05 | 1,00E+00 | 1,50E-03 |
| T cell costimulation GO:0031295 | 5 | 11,6 | 3,50E-05 | 1,00E+00 | 1,90E-03 |
| defense response to Gram-positive bacterium GO:0050830 | 5 | 11,6 | 4,90E-05 | 1,00E+00 | 2,40E-03 |
| antibacterial humoral response GO:0019731 | 4 | 9,3 | 1,50E-04 | 1,00E+00 | 6,80E-03 |
| T cell receptor signaling pathway GO:0050852 | 5 | 11,6 | 4,10E-04 | 1,00E+00 | 1,70E-02 |
| oxygen transport GO:0015671 | 3 | 7 | 5,70E-04 | 9,99E-01 | 2,10E-02 |
| intracellular estrogen receptor signaling pathway GO:0030520 | 3 | 7 | 1,10E-03 | 9,99E-01 | 3,80E-02 |

**Supplementary Table 5. A proteomic comparison between AML cell populations showing generally increased PI3K-Akt-mTOR phosphorylation versus patients showing divergent effects after exposure to insulin.** The results are presented as the most significant GO-terms when analyzing Biological Processes, and the table shows the GO-terms, the number of significantly altered proteins belonging to the term, the percent of altered proteins out of the total number of proteins belonging to the term, the p-value and the FDR.

| **GO-term** | **Gene Count** | **%** | **P-Value** | **Benjamini** |
| --- | --- | --- | --- | --- |
| Cellular Ketone Metabolic Process GO:0042180 | 34 | 0,9 | 2,10E-10 | 3,90E-07 |
| Organic Acid Metabolic Process GO:0006082 | 33 | 0,9 | 6,30E-10 | 4,00E-07 |
| Carboxylic Acid Metabolic Process GO:0019752 | 33 | 0,9 | 5,30E-10 | 5,00E-07 |
| Oxoacid Metabolic Process GO:0043436 | 33 | 0,9 | 5,30E-10 | 5,00E-07 |
| Oxidation Reduction Process GO:0055114 | 29 | 0,8 | 2,00E-06 | 5,30E-04 |
| Alcohol Metabolic Process GO:0006066 | 25 | 0,7 | 1,50E-07 | 7,20E-05 |
| Cellular Carbohydrate Metabolic Process GO:0044262 | 21 | 0,6 | 5,80E-06 | 1,20E-03 |
| Cellular Amino Acid And Derivative Metabolic Process GO:0006520 | 19 | 0,5 | 2,00E-05 | 2,40E-03 |
| Monocarboxylic Acid Metabolic Process GO:0032787 | 18 | 0,5 | 9,50E-06 | 1,60E-03 |
| Generation Of Precursor Metabolites And Energy GO:0006091 | 18 | 0,5 | 1,60E-05 | 2,30E-03 |
| Monosaccharide Metabolic Process GO:0005996 | 17 | 0,5 | 7,60E-07 | 2,90E-04 |
| Cellular Amino Acid Metabolic Process GO:0006520 | 17 | 0,5 | 9,70E-07 | 3,00E-04 |
| Nucleotide Metabolic Process GO:0009117 | 17 | 0,5 | 2,60E-05 | 2,90E-03 |
| Nucleoside Phosphate Metabolic Process GO:0006753 | 17 | 0,5 | 2,60E-05 | 2,90E-03 |
| Cellular Amine Metabolic Process GO:0044106 | 17 | 0,5 | 4,30E-05 | 4,20E-03 |
| Hexose Metabolic Process GO:0019318 | 14 | 0,4 | 1,70E-05 | 2,20E-03 |
| Cofactor Metabolic Process GO:0051186 | 14 | 0,4 | 2,00E-05 | 2,40E-03 |
| Glucose Metabolic Process GO:0006006 | 12 | 0,3 | 4,30E-05 | 4,00E-03 |
| Coenzyme Metabolic Process GO:0006732 | 12 | 0,3 | 4,30E-05 | 4,00E-03 |
| Monosaccharide Catabolic Process GO:0046365 | 10 | 0,3 | 2,20E-06 | 5,20E-04 |
| Alcohol Catabolic Process GO:0046164 | 10 | 0,3 | 6,80E-06 | 1,30E-03 |
| Cellular Carbohydrate Catabolic Process GO:0044275 | 10 | 0,3 | 1,00E-05 | 1,60E-03 |
| Glucose Catabolic Process GO:0006007 | 8 | 0,2 | 4,20E-05 | 4,40E-03 |
| Ribonucleoside Monophosphate Metabolic Process GO:0009161 | 6 | 0,2 | 4,60E-05 | 4,10E-03 |

**Supplementary Table 6. A phosphoproteomic comparison between AML cell populations showing generally increased PI3K-Akt-mTOR phosphorylation versus patients showing divergent effects after exposure to insulin.** The results are presented as the most significant GO-terms when analyzing Biological processes based on 77 genes included in the term Cellular Component-Intracellular Membrane-Bounded Organelle. The table shows the GO-term, the number of significantly different proteins belonging to the term, the percent of altered proteins out of the number of proteins belonging to the term, the p-value and the corrected FDR (Benjamini).

| **GO-Term** | **Gene count** | **%** | **P-Value** | **Benjamini** |
| --- | --- | --- | --- | --- |
| Primary metabolic process GO:0044238 | 114 | 54,3 | 4,70E-05 | 3,00E-03 |
| Cellular metabolic process GO:0044237 | 112 | 53,3 | 1,70E-05 | 1,70E-03 |
| Macromolecule metabolic process GO:0043170 | 106 | 50,5 | 2,70E-07 | 3,10E-05 |
| Cellular macromolecule metabolic process GO:0044260 | 102 | 48,6 | 3,50E-08 | 4,80E-06 |
| Cellular nitrogen compound metabolic process GO:0034641 | 82 | 39 | 9,30E-09 | 1,40E-06 |
| Nitrogen compound metabolic process GO:0006807 | 82 | 39 | 3,80E-08 | 4,70E-06 |
| Nucleobase, nucleoside, nucleotide and nucleic acid metabolic process GO:0006139 | 81 | 38,6 | 6,10E-10 | 1,40E-07 |
| Gene expression GO:0010467 | 73 | 34,8 | 3,10E-09 | 6,10E-07 |
| Regulation of macromolecule metabolic process GO:0060255 | 66 | 31,4 | 2,70E-05 | 2,20E-03 |
| Regulation of gene expression GO:0010468 | 64 | 30,5 | 1,30E-06 | 1,40E-04 |
| Regulation of cellular biosynthetic process GO:0031326 | 61 | 29 | 3,60E-05 | 2,70E-03 |
| Regulation of biosynthetic process GO:0009889 | 61 | 29 | 4,40E-05 | 2,90E-03 |
| Regulation of macromolecule biosynthetic process GO:0010556 | 60 | 28,6 | 2,10E-05 | 1,80E-03 |
| Regulation of transcription GO:0006355 | 55 | 26,2 | 6,30E-05 | 3,90E-03 |
| RNA metabolic process GO:0016070 | 39 | 18,6 | 8,00E-11 | 2,20E-08 |
| Organelle organization GO:0006996 | 36 | 17,1 | 1,90E-05 | 1,70E-03 |
| RNA processing GO:0006396 | 34 | 16,2 | 4,00E-14 | 2,70E-11 |
| mRNA metabolic process GO:0016071 | 28 | 13,3 | 1,20E-13 | 5,50E-11 |
| RNA splicing GO:0008380 | 27 | 12,9 | 1,70E-15 | 2,30E-12 |
| mRNA processing GO:0006397 | 26 | 12,4 | 2,50E-13 | 8,50E-11 |
| Chromosome organization GO:0051276 | 19 | 9 | 3,90E-05 | 2,80E-03 |
| RNA splicing, via transesterification reactions GO:0000375 | 15 | 7,1 | 7,50E-09 | 1,30E-06 |
| RNA splicing, via transesterification reactions with bulged Adenosine as nucleophile GO:0000377 | 15 | 7,1 | 7,50E-09 | 1,30E-06 |
| Nuclear mRNA splicing, via spliceosome GO:0000398 | 15 | 7,1 | 7,50E-09 | 1,30E-06 |
